# Therapeutic Potential of Antiviral Peptides against the NS2B/NS3 Protease of Zika Virus

**DOI:** 10.1021/acsomega.3c04903

**Published:** 2023-09-13

**Authors:** Md. Shahadat Hossain, Md. Tanjil Islam Shovon, Md. Rafid Hasan, Fuad Taufiqul Hakim, Mohammad Mehedi Hasan, Sadia Afrose Esha, Sabiha Tasnim, Md. Shahoriar Nazir, Fahmida Akhter, Md Ackas Ali, Mohammad A. Halim

**Affiliations:** † Division of Infectious Diseases and Division of Computer-Aided Drug Design, The Red-Green Research Center, BICCB, Tejgaon 1215, Dhaka, Bangladesh; ‡ Department of Chemistry and Biochemistry, Kennesaw State University, Kennesaw, Georgia 30144, United States

## Abstract

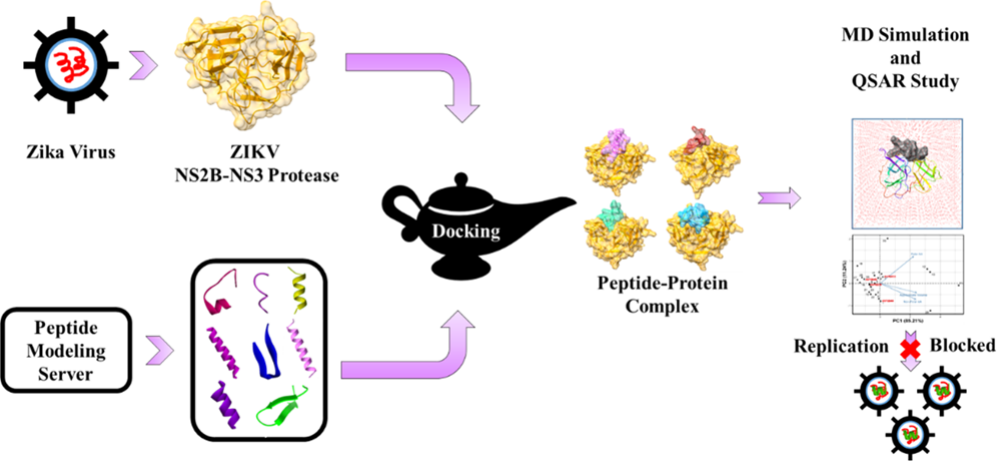

The NS2B/NS3 protease is highly conserved among various proteases of the Zika virus, making it an important therapeutic target for developing broad-spectrum antiviral drugs. The NS2B/NS3 protease is a crucial enzyme in the replication cycle of Zika virus and plays a significant role in viral maturation and assembly. Inhibiting the activity of this protease can potentially prevent viral replication, making it an attractive target for developing therapies against Zika virus infection. This work screens 429 antiviral peptides in comparison with substrate peptide against the NS2B/NS3 of Zika virus using molecular docking and molecular dynamics (MD) simulation. Based on the docking screening, MD simulation conducted for the best four peptides including AVP0239, AVP0642, AVP0660, and AVP2044, could be effective against NS2B/NS3. These results were compared with the control substrate peptide. Further analysis indicates that AVP0642 and AVP2044 are the most promising candidates. The interaction analysis showed that the catalytic site residues including His51, Asp75, Ser135 and other non-catalytic residues such as Asp129, Asp83, and Asp79 contribute substantial interactions. Hydrogen bonds (41%) and hydrophobic interactions (33%) are observed as the prominent non-covalent interaction prompting the peptide–protein complex formation. Furthermore, the structure–activity relationship (SAR) illustrates that positively charged (Lys, Arg) residues in the peptides dominate the interactions. This study provides the basis for developing novel peptide-based protease inhibitors for Zika virus.

## Introduction

The Zika virus is a mosquito-borne pathogen that belongs to the Flaviviridae family and genus Flavivirus.^1^ Other members of this family include Dengue, West Nile, Japanese encephalitis, and yellow fever virus.^2^ Zika virus infection is a dangerous emerging illness that has drawn significant attention from a public health perspective.^3^ This infection can cause mild symptoms such as fever, rash, arthritis, headache, and conjunctivitis.^4^ However, during pregnancy, Zika virus infection can lead to severe neurodevelopmental damage, ocular defects, cerebral calcifications, and fetal microcephaly.^5^ The rapid spread of these infections in the regions of the world has raised international concern about the disease. As a result, the World Health Organization (WHO) highlighted the urgent need in developing effective drugs to combat this emerging threat.^3^ Despite the attention that Zika virus has received, there still needs to be more knowledge and expertise about this virus.

In 1947, Zika virus was accidentally isolated in Uganda’s Zika jungle and later, discovered in Aedes africanus mosquitoes in 1948.^6^ Humans are considered as the pathogen’s amplifying host,^7^ while Aedes mosquitoes serve as the pathogen’s vectors.^8^ The first human case of Zika virus infection was identified in Uganda and the United Republic of Tanzania in 1952.^9^ In 1954, Zika virus was confirmed to be causing infection linked to jaundice epidemics in Eastern Nigeria.^10^ During the 1960s and 1980s, seven human instances of Zika virus infection were discovered in Asia (India, Indonesia, Malaysia, Pakistan) and Africa.^11^ The Yap Islands of Micronesia had the first outbreak of Zika virus, with around 5000 cases reported in the year 2007,^12^ which spread over the other Pacific regions.^13−15^ In 2016, autochthonous Zika virus transmission (mosquito-borne infection) was confirmed in 35 countries and territories, including Bolivia, Brazil, Colombia, Cuba, Dominica, Ecuador, French Guiana, Honduras, Korea, Marshall Islands, Mexico, Paraguay, Saint Martin, Saint Vincent and the Grenadines, Tonga and Tobago, and Venezuela.^16^ Although *Aedes aegypti* is the primary epidemic vector, the virus has been isolated from a variety of Aedes species that are likely implicated in Zika virus transmission to people.^17^

Zika virus is an enveloped, single-stranded, positive-sense RNA virus with icosahedral symmetry and a non-segmented genome of 10 794 nucleotides encoding 3419 amino acids.^18^ The genome’s linear RNA encodes a polyprotein in the “long open reading frame,” which includes all structural protein genes at the 5′ end and nonstructural protein genes at the 3′ end having the following protein expression: 5′ C-prM-E-NS1-NS2A-NS2B/NS3-NS4A-NS4B-NS5 3′.^19^ The open reading frame of the Zika virus genome is divided into three structural proteins including Capsid (C), precursor to Membrane (prM), and Envelope (E) and seven nonstructural (NS) proteins [NS1, NS2A, NS4A, NS2B, NS4B, NS3, and NS5].^20,16^ Among the viral target components, the NS2B/NS3 protein is a viral protease enzyme that is essential for the replication of the Zika virus. The NS2B protein functions as a cofactor that is required for the NS3 protease domain to achieve its optimal conformation and activity. Together, the NS2B/NS3 protease complex cleaves the viral polyprotein into individual proteins that are necessary for viral replication, including the viral capsid protein and the RNA-dependent RNA polymerase.^21,22^ The NS2B/NS3 protease also plays a critical role in viral maturation and assembly,^23,24^ making it an attractive target for the development of antiviral therapies against Zika virus infection. This complex is also considered a drug target because it is conserved among many different viruses, including hepatitis C virus and dengue virus, and others.^21^

While small molecules have been a cornerstone of drug development for decades, there are several limitations associated with their use as therapeutics against viral infections.^25−28^ Small molecules may have limited cellular absorption due to their physicochemical properties and may not reach their intracellular targets at effective concentrations. Their inability to penetrate across cell membranes restricts their clinical applicability for drug development,^29,30^ particularly for viruses that replicate intracellularly. Furthermore, small molecules may exhibit off-target effects, leading to toxicity and potentially compromising the efficacy of the therapy.^31^ This is where antiviral peptides have an advantage. Despite the fact that small molecules are the most common type of antiviral, peptides combine the benefits of both protein therapies and small molecules.^32^ Peptide-based medications offer several advantages as drug therapies, including their high specificity and selectivity, which can reduce the risk of off-target effects and improve therapeutic outcomes.^32,33^ Peptides also have a dynamic binding nature, allowing them to adapt to different conformations and interact with multiple targets, making them versatile drug candidates.^32^ Additionally, peptides are generally non-immunogenic that improve safety and reduce the risk of adverse reactions. These advantages have led to a growing interest in peptide-based drugs, with more than 170 peptides currently in active clinical development and many more in preclinical studies.^32,34^ Despite the advantages, peptide therapeutics have some pharmacokinetic issues such as instability in biological systems due to the presence of proteolytic enzymes, impermeability to biological membranes, and poor oral bioavailability. Stapling, incorporating unnatural amino acids, and modifying N and C terminals by acetylation, and amidation can significantly improve the peptide serum stability by restricting the proteolytic degradation.^35−37^ Moreover, conjugating the designed peptide with cell-penetrating peptide (CPP) and glycosylation such as N- and O-linked glycosylation can solve the problems associated with poor permeability and oral bioavailability.^38,39^

This study involved an in-depth computational analysis to evaluate the antiviral potential of 429 peptides against the Zika virus NS2B/NS3. Using a combined molecular docking approach, virtual screening was used to find potential peptides for Zika virus NS2B/NS3. Molecular dynamics (MD) simulations were used to evaluate the selected peptides. This research aims to provide promising insights into the design and development of antiviral peptides for Zika virus therapeutic intervention.

## Results and Discussion

### Peptides Binding Affinity and Interaction

In this study, an initial screening of 429 antiviral peptides and one control substrate peptide (Table S1) were conducted using the HawkDock server to investigate their binding affinities and interactions with the NS2B/NS3 protease of the Zika virus. A four-amino acid peptide (RKKR)-based substrate [29], which showed the specificity to the NS2B/NS3 protease, was used as a control peptide. The HawkDock server was used to dock all the peptides against the NS2B/NS3 of the Zika virus, the binding pocket region was not specified to obtain more accurate results. Additionally, multiple docking was conducted using PatchDock, FireDock, and HADDOCK to further investigate the peptides’ binding modes (Table S2). The best complexes from the HawkDock server were then selected for molecular dynamics (MD) simulation, based on the HawkDock score and the observed binding mode. The HawkDock scores for the selected peptides were found to be in the range of −85.17 to −2.17 (Table S2). According to these scores, the top 10 best poses of the NS2B/NS3–AVP2044, NS2B/NS3-AVP2046, NS2B/NS3-AVP0018, NS2B/NS3-AVP0623, NS2B/NS3-AVP2045, NS2B/NS3-AVP0641, NS2B/NS3–AVP0660, NS2B/NS3-AVP1821, NS2B/NS3–AVP0239, NS2B/NS3–AVP0642, and NS2B/NS3–substrate–peptide complexes were selected for MD simulation (Table 1).

**Table 1:** BFE and Binding Scores of Top 10 Peptides and the Substrate Peptide against the ZIKV NS2B-NS3 Obtained From Hawkdock

AVPid	Hawkdock score	Hawkdock BFE (kcal/mol)
AVP0018	–69.08	–69.33
AVP0239	–57.4	–53.59
AVP0623	–68.53	–69.29
AVP0641	–64.78	–64.72
AVP0642	–57.22	–60.42
AVP0660	–60.08	–57.38
AVP1821	–58.53	–56.55
AVP2044	–85.17	–84.19
AVP2045	–67.1	–65.31
AVP2046	–77.2	–78.87
substrate peptide	–39.68	–39.68

### Molecular Dynamics (MD) Simulation

**Figure 1:**
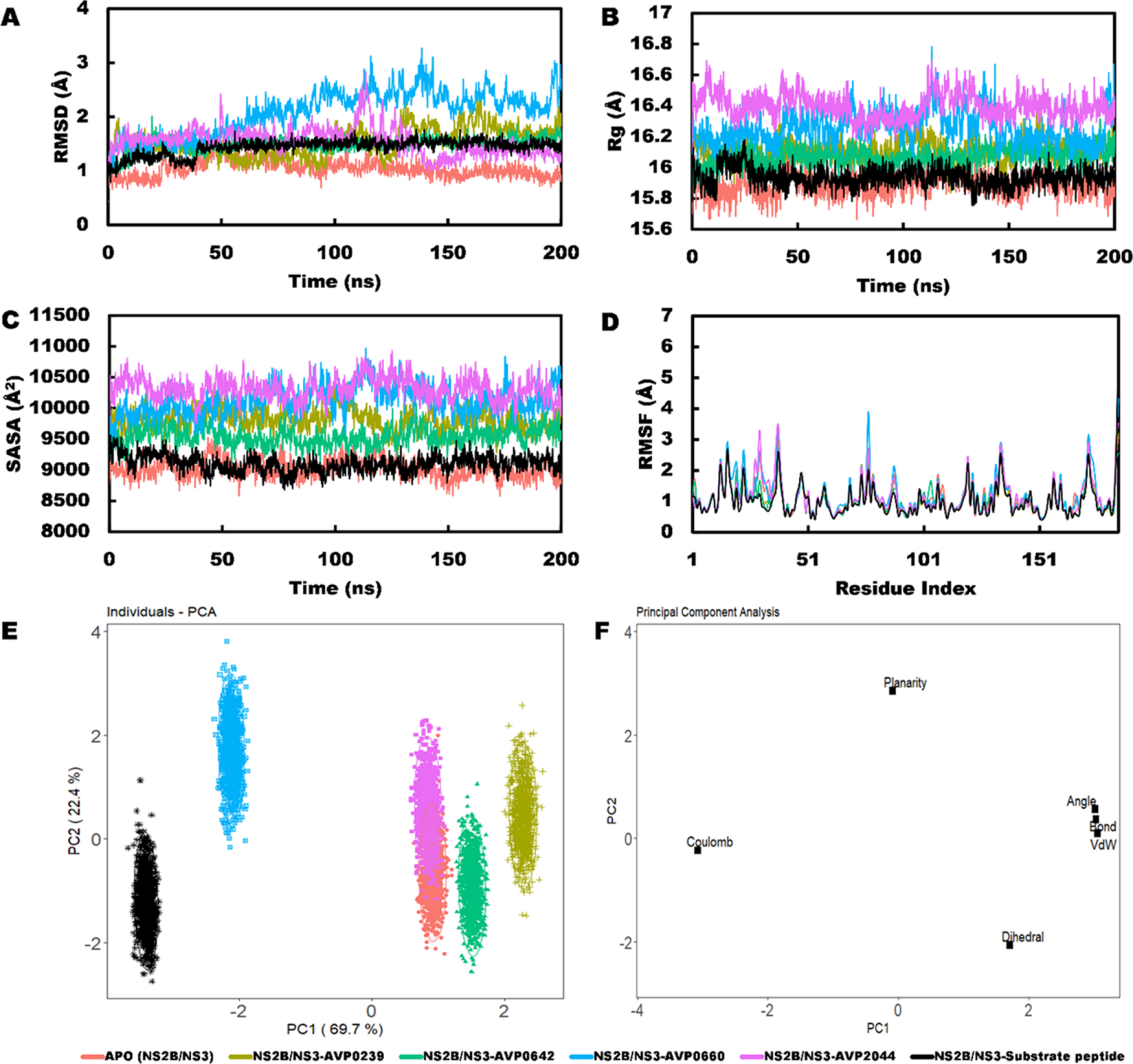
Molecular dynamics simulation. (A) Root-mean squared deviation (RMSD); (B) radius of gyration (Rg); (C) solvent-accessible surface area (SASA); (D) root-mean-squared fluctuation (RMSF); (Sequence 1-37, NS2B and 38-185, NS3); (E) scores plot; and (F) loading plot of top four peptides–NS2B/NS3 complexes and a substrate peptide over 200 ns simulation.

**Figure 2:**
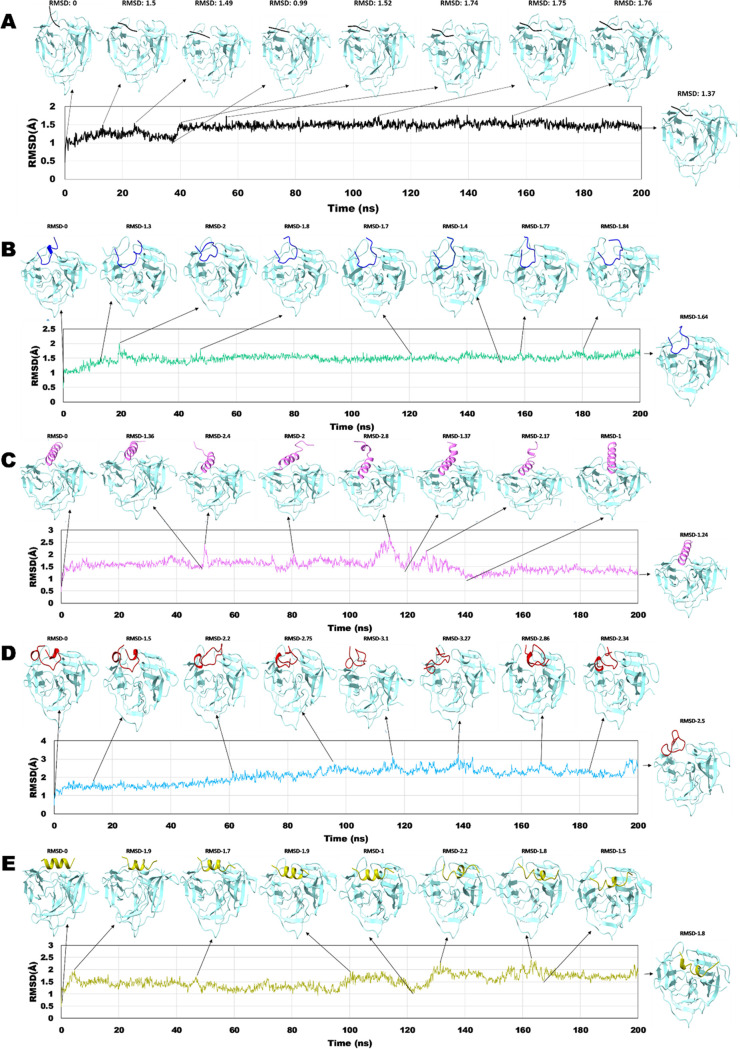
Representative snapshots with RMSD. NS2B/NS3 (Middle Blue Green); (A) NS2B/NS3–substrate peptide; (B) NS2B/NS3–AVP0642; (C) NS2B/NS3–AVP2044; (D) NS2B/NS3–AVP0660; (E) NS2B/NS3–AVP0239; over 200 ns MD simulation.

Initially, MD simulations were performed to compare the effect of peptide presence on the stability of the top 10 HawkDock complexes and the NS2B/NS3 apo. Finally, four peptides were selected for long-term simulations based on RMSD, Rg, and SASA values. MD simulation was also conducted for the control substrate peptide. The results showed that the NS2B/NS3–AVP0642 and substrate peptide complexes had the most stable RMSD profile, followed by NS2B/NS3–AVP2044, NS2B/NS3–AVP0239, and NS2B/NS3–AVP0660 complexes (Figure 1A). The RMSD values for both the NS2B/NS3–AVP0642 and NS2B/NS3–substrate peptide complexes were slightly higher than that of the apo protein, but their standard deviations were lower. Initially, the RMSD value of the NS2B/NS3–substrate peptide complex was lower than that of NS2B/NS3–AVP0642. However, after 35 ns, it underwent an inward movement and overlapped with the NS2B/NS3–AVP0642 complex, which was maintained in the subsequent period. The NS2B/NS3–AVP2044 complex fluctuated between 100 and 140 ns before becoming stable, while the NS2B/NS3–AVP0239 complex was stable at the beginning but fluctuated after 90 ns. The NS2B/NS3–AVP0660 complex had the highest fluctuation values and exceeded an RMSD value of 3 Å at its maximum. MD snapshots revealed that these peptides occupied the binding interface and remained as stable complexes throughout the simulation period (Figure 2A–E). The NS2B/NS3–AVP0642 and NS2B/NS3–substrate peptide complexes also exhibited a stable Rg profile. In this regard, the NS2B/NS3–substrate complex merged with the apo protein, while the NS2B/NS3–AVP0642 complex experienced a slightly higher Rg value. The NS2B/NS3–AVP0239 and NS2B/NS3–AVP0660 complexes had slightly increasing trends and fluctuated after 100 ns. The NS2B/NS3–AVP2044 complex had a higher Rg value and fluctuated more between 100 and 135 ns before becoming stable, but it still had a higher Rg value (Figure 1B). A nearly identical trend was also observed in the solvent-accessible surface area (SASA) values. The NS2B/NS3–substrate peptide complex merged with the apo protein, while the NS2B/NS3–AVP0642 complex exhibited a slightly higher value (Figure 1C). Other complexes experienced higher SASA value compared to these complexes. RMSF values for all complexes were similar except for the NS2B/NS3–AVP0660 and NS2B/NS3–AVP2044 complexes (Figure 1D). Overall, the NS2B/NS3–substrate peptide, NS2B/NS3–AVP0642, and NS2B/NS3–AVP2044 complexes demonstrated consistency with RMSDCα, Rg, and SASA values, making them the most stable among the complexes studied.

### Peptide Conformational Transitions: Unveiling RMSD Shifting

#### NS2B/NS3–Substrate Peptide

An initial inward movement is observed in the substrate peptide; however, there is no significant change noted in its structure. After 35 ns, a noticeable increase in RMSD is observed due to the substrate peptide’s inward movement. After this transition, it achieves a favorable position and remains stable throughout the simulation period despite substantial dynamic fluctuations within the binding pocket (Figure 2A).

#### NS2B/NS3–AVP0642

In this complex, the structural and rotational changes of the peptide within the binding pocket resulted in significant fluctuations in RMSD at certain time points. At 18.4 ns, the peptide transitioned from a helical shape to a coil and maintained this conformation until the end of the simulation. At 19.4 ns, the peptide moved outward from the binding pocket in a highly compact shape, resulting in a high RMSD of 2 (Figure 2B).

#### NS2B/NS3–AVP2044

Throughout the MD simulation, the peptide mostly maintained its native a-helix shape while residing in the binding pocket. However, at certain time points, the terminal of the peptide transitioned to a coil shape, resulting in an increase in RMSD. At 49.4 ns, the terminal of the peptide shifted to a coil shape, resulting in an RMSD of 2.4. Similarly, at 80.6 ns, a rotational change in the peptide resulted in an RMSD of 2. At 113.8 ns, the peptide adopted a coil shape and rotation, resulting in an RMSD of 2.8. Finally, at 140.2 ns, the peptide returned to its native helical structure and moved inward toward the binding pocket, resulting in an RMSD of 1. This pattern remained relatively consistent until the end of the simulation (Figure 2C).

#### NS2B/NS3–AVP0660

The initial structure of the peptide was a combination of helix and coil, which eventually transitioned to a coil at the endpoint, resulting in fluctuations in RMSD. At 65.5 ns, the peptide underwent a structural transition from a helix to a coil and was rotated from its initial position, resulting in an RMSD of 2.2. At 96.7 ns, the peptide adopted a compact shape and moved outward from the binding pocket, resulting in an RMSD of 2.75. However, from 116 to 138.5 ns, the peptide moved further away from the protein binding pocket, resulting in an RMSD value greater than 3 (Figure 2D).

#### NS2B/NS3–AVP0239

During the simulation, the peptide remained within the binding pocket, with fluctuations in RMSD observed at certain time points. At 4.5 ns, the peptide terminal transitioned from a helix to a coil, which persisted until 122 ns. Due to the upward movement of the peptide from the binding pocket, an increase in RMSD was observed at 4.6, 46.8, and 100.9 ns. Finally, at 131.4 ns, the peptide transitioned to a coil and RMSD increased to 2.2, although it subsequently rapidly decreased as the peptide moved inward toward the binding pocket. Notably, the highest fluctuation in RMSD was observed at 131 ns, with a value of 2.2 (Figure 2E).

### Principal Component Analysis

A comparative PCA model was built using the last 100 ns of MD simulation to gain a better understanding of structural and energy changes in the ligand–protein complexes. The model included the coulomb energy, angle, bond distance, dihedral, planarity, and VdW energies for five training data sets (apo and four ligand–apo complexes). The results showed that the first two PCs explained 92.1% of the variance, with PC1 contributing 69.7% and PC2 contributing 22.4%. The score plot indicated that the apo and NS2B/NS3–AVP0239, NS2B/NS3–AVP0642, NS2B/NS3–AVP2044 complexes had a similar pattern, while the NS2B/NS3-AVP0660 and NS2B/NS3–substrate peptide complexes were distinct (Figure 1E). Dihedral, angle, VdW, angle, and bond energy positively correlated with PC1, while Coulomb and planarity negatively correlated. Dihedral and Coulomb positively correlated with PC2, while other energies negatively correlated (Figure 1F). Coulomb was identified as a factor of shifting NS2B/NS3–substrate peptide, while planarity is responsible for NS2B/NS3-AVP0660 shifting. It is commonly observed that the clustering patterns obtained from PCA analysis bear a similarity to those obtained from QSAR analysis especially for NS2B/NS3AVP0239 and NS2B/NS3–AVP0642 complexes (Figure 7).

### Binding Free Energies

**Figure 3:**
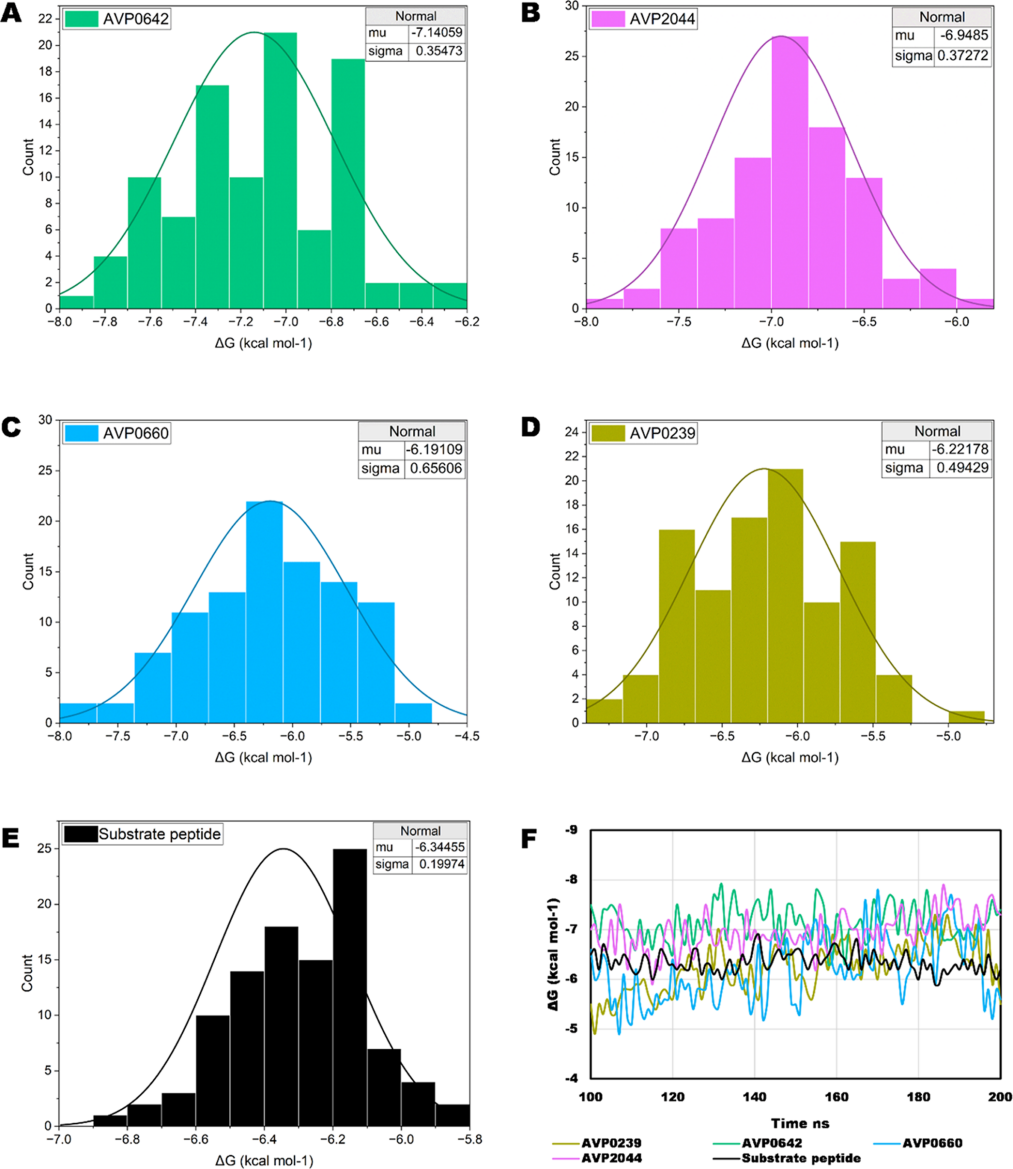
Binding free energy distribution of complexes in kcal/mol. (A) NS2B/NS3–AVP0642; (B) NS2B/NS3–AVP2044; (C) NS2B/NS3–AVP0660; (D) NS2B/NS3–AVP0239; (E) NS2B/NS3–substrate peptide; (F) comparison of binding free of last 100 ns simulation snapshots.

The binding affinities of four selected complexes along with the substrate peptide were evaluated using the PRODIGY server, which revealed that these complexes exhibited consistently negative values throughout the simulation period. This suggests that the complexes exhibit strong binding and do not dissociate from the binding pocket (Figure 3F). This was further supported by the analysis of RMSD values with their snapshots (Figure 2A–E). Upon evaluating the average binding free energy values, AVP0642 and AVP2044 exhibited notably higher binding affinity compared to the substrate peptide, while AVP0239 and AVP0660 displayed comparatively lower affinities (Figure 3A–E). A comparison of the binding affinities of the AVP0642 and AVP2044 peptides revealed that they have similar binding strengths, with an average value of approximately −7 kcal/mol. Both peptides maintained stable interactions over the simulation period (Figure 3A,B). In contrast, the AVP0239 and AVP0660 peptides exhibited lower binding affinities, which are slightly lower than the substrate peptide, with an average value of around −6 kcal/mol (Figure 3C–E). This similarity in behavior was also reflected in the stable profiles of RMSD, radius of gyration (Rg), and solvent-accessible surface area (SASA) of the complexes (Figure 1A–C).

### Residue Interaction Analysis

Interactions between the NS2B/NS3 with AVP0642, AVP2044, AVP0239, AVP0660 peptides, and the control substrate were thoroughly analyzed over the course of the last 100 ns of MD simulations. Notably, the outcomes unveiled profound interactions within the NS2B/NS3–substrate peptide complex, where specific residues, namely, Asp83, Gly153, Val155, and Tyr161 in the NS2B/NS3 region, exhibited significant interactions. Moreover, this complex demonstrated a noteworthy affinity toward catalytic residues such as Asp75 and His51 (Figure 5B). Detailed examination of the final nanosecond interactions further substantiated the peptide’s strong entrenchment within the binding pocket, facilitating crucial interactions with catalytic and other adjacent residues (Figure 5A). Among the substrate peptide residues, the key contributors were identified as Arg4 and Lys3.

**Figure 4:**
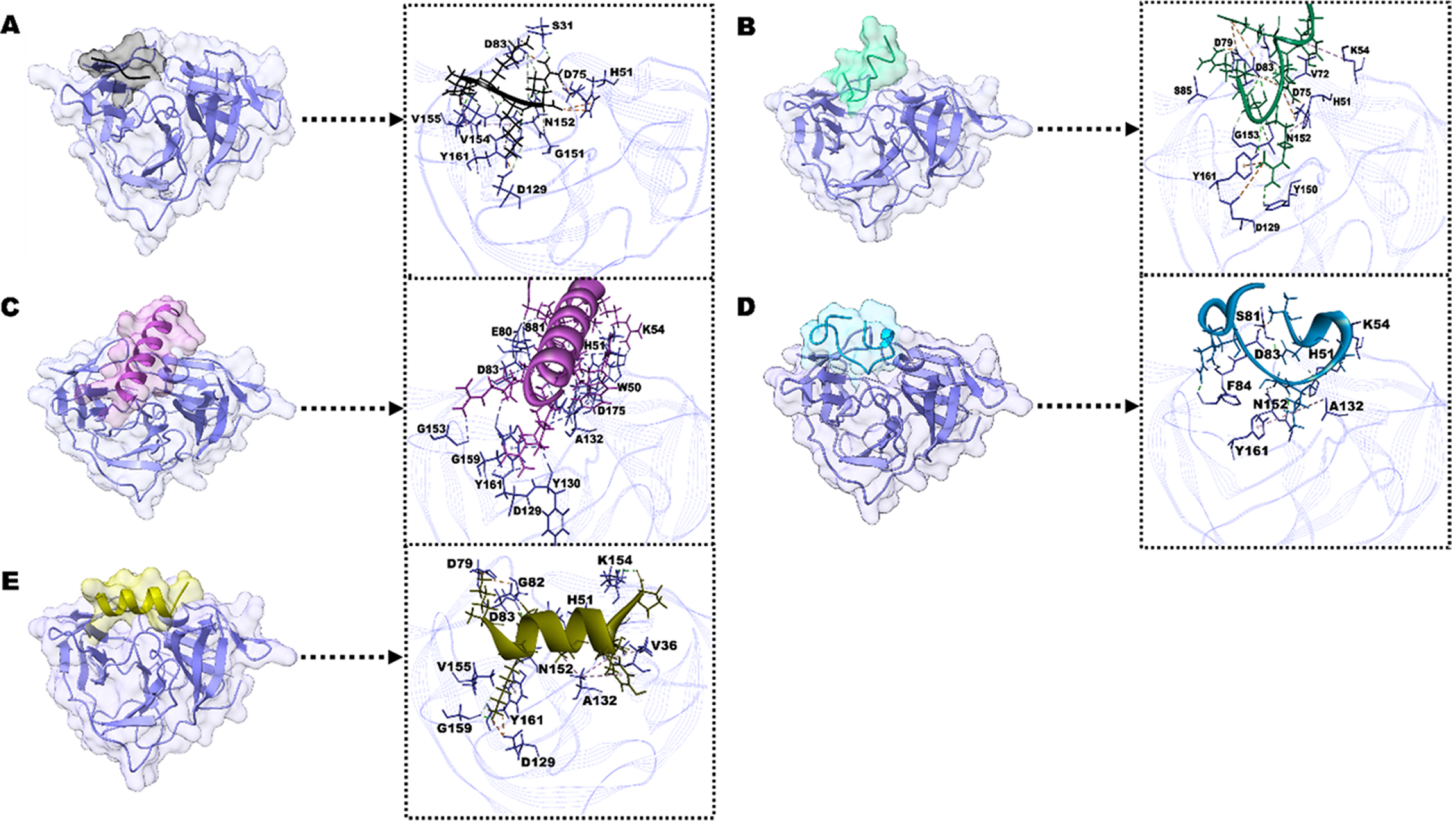
Peptide–protein interaction closeview. (A) NS2B/NS3–substrate peptide; (B) NS2B/NS3–AVP0642; (C) NS2B/NS3–AVP2044; (D) NS2B/NS3–AVP0660; (E) NS2B/NS3–AVP0239; of final snapshots at 200 ns.

Within the NS2B/NS3–AVP0642 complex, major interactions were observed involving NS2B/NS3 residues, including Asp83, Asp79, Asp129, Tyr161, and Val155. Notably, at the end of simulation, the peptide was ensconced within the binding pocket, promoting significant engagement with catalytic residues. This phenomenon is depicted in Figures 4B and 6B. A closer picture of the AVP0642 peptide highlighted the repeated interactions involving Arg6, Arg4, Arg7, Arg11, and Thr2 (Figure 6A).

In NS2B/NS3–AVP2044 complex, significant interactions were elucidated, notably featuring Asp129, Asp83, Val87, Ala132, and Asp79 within the NS2B/NS3 region (Figure S1B). Despite a partial burial within the binding pocket during the simulation, this complex showcased remarkable interactions with catalytic residues, as illustrated in Figure 4C. Within the AVP2044 peptide, the residues contributing significant interactions were identified as Arg13, Arg6, Arg2, and Arg9 (Figure S1A).

In the NS2B/NS3–AVP0239 complex, noticeable interactions surfaced, involving NS2B/NS3 residues Asp83, His51, and Ala132. Similarly, within the NS2B/NS3–AVP0660 complex, NS2B/NS3 residues, Asp83, Asp129, and Tyr161, were engaged in extensive interactions, as evidenced by Figures S2B and S3B. It is worth noting that these two complexes exhibited interactions solely with a single catalytic residue throughout the simulation period, a fact corroborated by the detailed analysis of final nanosecond interactions (Figure 4D,E).

**Figure 5:**
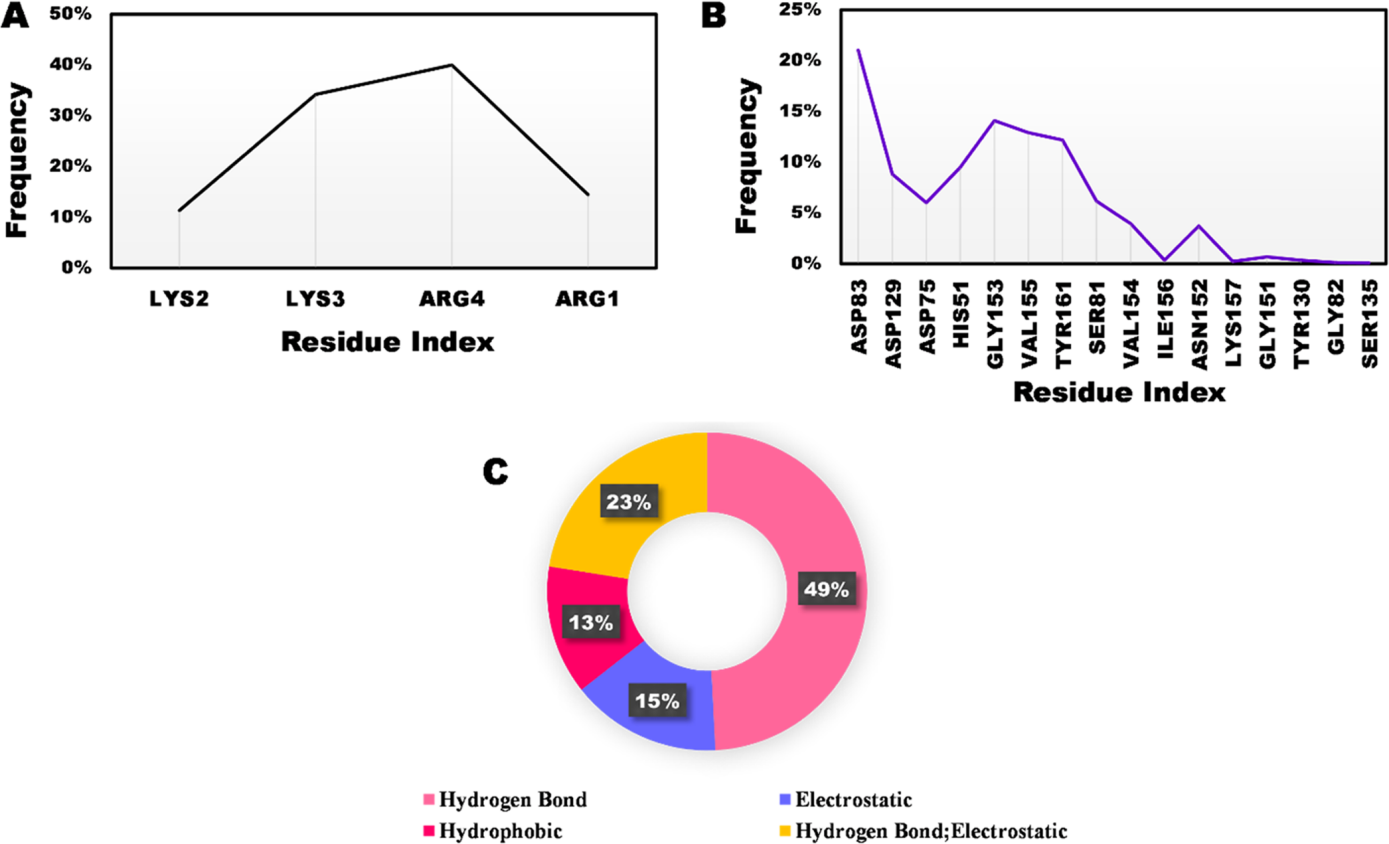
NS2B/NS3–substrate peptide complex. (A) Interacting substrate peptide residues; (B) interacting NS2B/NS3 residues; (C) distribution of non-covalent interactions; over 200 ns MD simulation.

**Figure 6:**
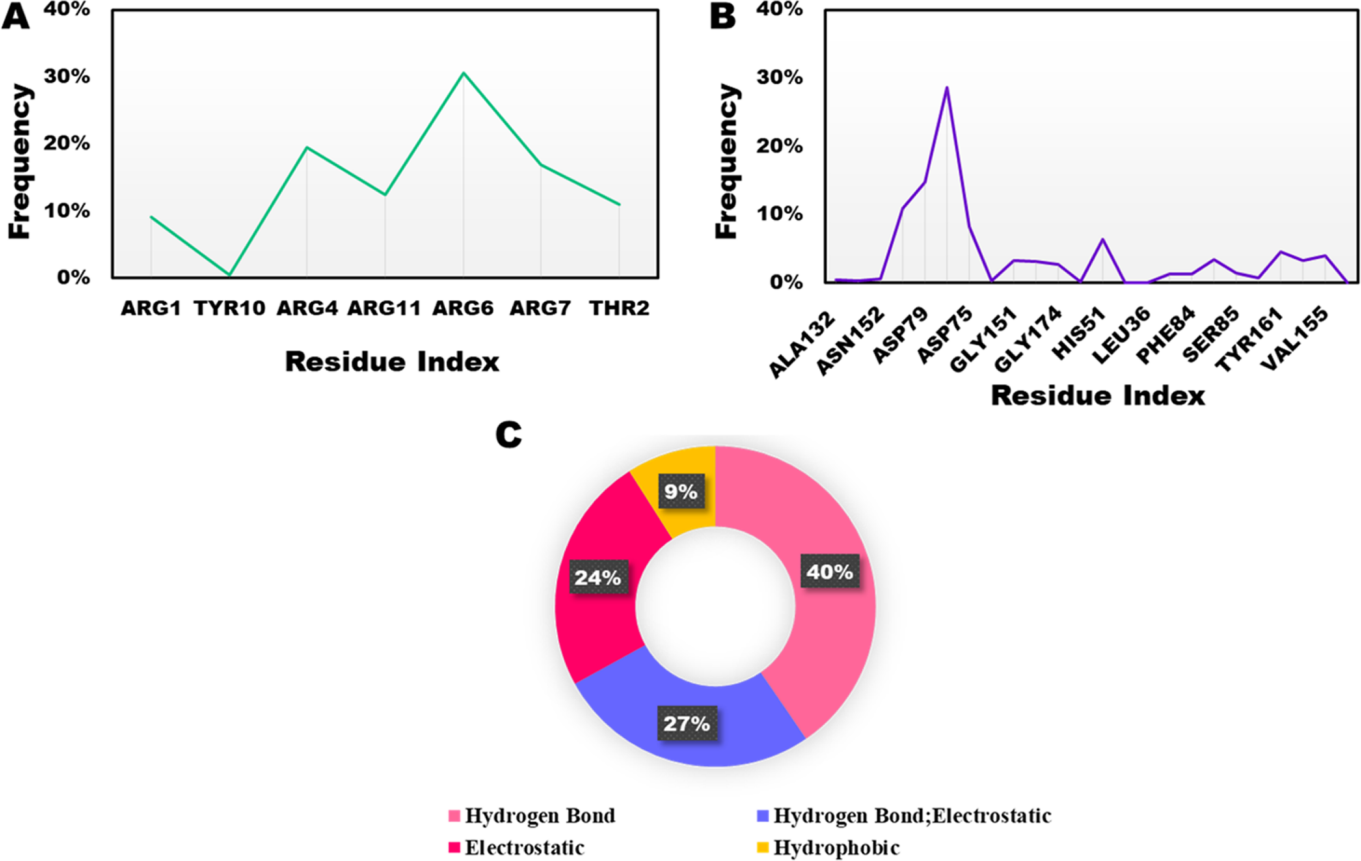
NS2B/NS3–AVP0642 complex. (A) Interacting AVP0642 residues; (B) interacting NS2B/NS3 residues; (C) distribution of non-covalent interactions; over 200 ns MD simulation.

In the NS2B/NS3–substrate complex, the interaction profile was dominated by hydrogen bonding and hydrogen–electrostatic interactions. Further contributing to the intricate network, electrostatic and hydrophobic forces accounted for 15 and 13%, respectively, as illustrated in Figure 5C. The trajectory of interactions unveiled hydrogen bonding as the principal contributor in the NS2B/NS3–AVP0642 complex, encompassing 40% of the dynamic interplay throughout the simulation. Simultaneously, hydrogen–electrostatic interactions contributed 27%, while electrostatic and hydrophobic contributions balanced the ensemble with notable shares of 24% and 9%, respectively, as depicted in Figure 6C. Hydrogen bonding also played an important role (39%) in NS2B/NS3–AVP2044 complex over the course of the simulation. Hydrogen–electrostatic and electrostatic interactions contributed 22% each, while hydrophobic interactions contributed 18% as shown in Figure S1C. Similar trends are also observed in NS2B/NS3–AVP0660 and NS2B/NS3–AVP0239 complexes.

### Structure–Activity Relationship

**Figure 7:**
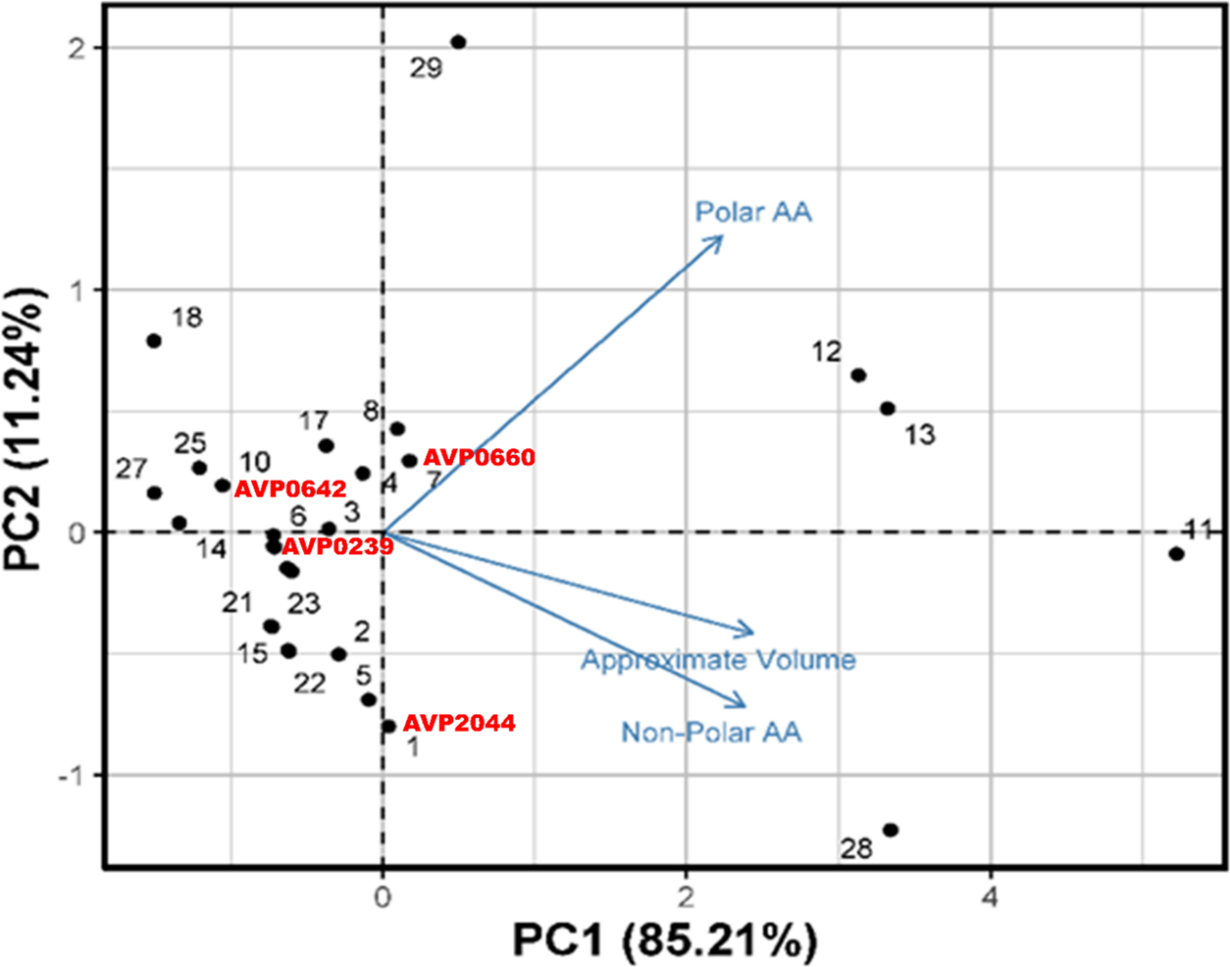
Biplot of the selected 30 high binding affinity peptides clustered based on four peptide properties.

Multiple linear regression (MLR) was employed to identify the major predictors of HawkDock binding scores for the top 30 protein–peptide complexes. Relevant predictors including the number of positively charged amino acids, number of negatively charged amino acids, number of polar amino acids, number of non-polar amino acids, theoretical pI, net charge at pH 7, GRAVY, and approximate volume were used in the analysis. The results indicated that non-polar amino acids, polar amino acids, and approximate volume were the major predictors of the score for HawkDock. Among them, the number of non-polar amino acids had the greatest impact. A positive correlation was observed between the HawkDock score and approximate volume, meaning that as the approximate volume increases, the interactions become stronger. Conversely, a negative correlation was observed between the number of non-polar and polar amino acids and the HawkDock score, indicating that as the number of these amino acids increases, the interactions become weaker (Table S3). A principal component analysis (PCA) was also conducted based on the three significant predictors identified by the MLR analysis to explore potential peptide recognition. The first principal component (PC1) represented 85.21% of the variability and was loaded with approximate volume and number of non-polar amino acids. The second component (PC2) was loaded with the number of polar amino acids and represented 11.24% of the variability (Figure 7). The PCA score plot revealed that peptides with similar properties formed clusters, such as AVP0239 and AVP0642, which align with the RMSD and RG profiles of these complexes. Additionally, the energy score plot showed that AVP0239, AVP0642, and AVP0660 formed a distinct group, which was also reflected in the PCA analysis of the molecular dynamics data. This suggests a correlation between the peptide properties and the dynamics of these complexes (Figures 1A,B,E and 7).

## Conclusions

The aim of this study was to identify effective peptide-based inhibitors of the Zika virus NS2B/NS3 protease. A total of 429 antiviral peptides were screened against the NS2B/NS3 of the Zika virus, and four of the most promising candidates were further studied through molecular dynamics simulation. Our comprehensive investigation into the interactions between peptide inhibitors and the Zika virus NS2B/NS3 protease has yielded valuable insights that contribute significantly to the understanding of potential therapeutic strategies. Through a comprehensive molecular dynamics simulations, residue interaction assessments, binding free energy evaluations, and structure–activity relationship elucidation, we have gained a profound understanding of the behavior, stability, and binding affinities of four promising peptide inhibitors: AVP0239, AVP0642, AVP0660, and AVP2044. The MD simulations unveiled dynamic shifts and conformational transitions within the peptide–protease complexes, offering insights into their stability profiles. Notably, AVP0642 has emerged as a standout candidate that remained stable within the binding pocket, displaying interactions with catalytic residues (His51 and Asp75) similar to those observed with the substrate peptide. Moreover, AVP0642 exhibited a remarkably stable RMSD profile, emphasizing its potential as a promising inhibitor. Its structural and rotational changes within the binding pocket remained within acceptable ranges, making it a highly promising inhibitor candidate. Furthermore, our exploration of residue-specific interactions shed light on the intricate choreography of molecular interplays within the complexes. The residues that have been identified—Asp83, Tyr161, and Val155—and their consistent binding with all complexes could prove invaluable for designing future protease inhibitors. Binding free energy assessments reinforced the stability of the complexes, with AVP0642 and AVP2044 displaying notably higher binding affinities compared to the substrate peptide. In summary, our study provides a comprehensive framework for understanding the interactions between peptide inhibitors and the Zika virus NS2B/NS3 protease. AVP0642 emerges as a highly promising candidate, demonstrating stability, strong interactions, and favorable binding affinities. Various staple analogues will be designed based on the best candidate peptides obtained from this study and tested their biological efficiency with protease and cell-based assays.

## Methods

### Molecular Docking

From the antiviral peptide database (AVPdb), 429 peptides with promising experimental evidence against various clinically significant viruses were chosen.^40^ A natural peptide substrate was also selected as a control . The selected peptides and the substrate peptide were modeled by PEP-FOLD^41^ and the Swiss model.^42^ Peptides containing less than 50 residues were primarily designed by PEP-FOLD, and for the rest of the cases, the Swiss model was used. The crystal structure of Zika virus NS2B/NS3 was retrieved from the RCSB Protein Data Bank (PDB ID: 5GPI).^43^ Initially, the crystal protein structure was docked against the selected peptide models utilizing multiple molecular docking web servers. Here, molecular dockings were performed by PatchDock,^44^ FireDock,^45,46^ and HADDOCK^47^ to check the best binding mode. Finally, blind docking was conducted by HawkDock server,^48^ and best complexes were finalized for MD simulation according to the HawkDock score.

### Molecular Dynamics (MD) Simulation

Initially, 25 ns MD simulation of the top 10 HawkDock complexes (NS2B/NS3–AVP2044, NS2B/NS3-AVP2046, NS2B/NS3-AVP0018, NS2B/NS3-AVP0623, NS2B/NS3-AVP2045, NS2B/NS3-AVP0641, NS2B/NS3–AVP0660, NS2B/NS3-AVP1821, NS2B/NS3–AVP0239, and NS2B/NS3–AVP0642) were performed to find out the relatively stable protein–peptide complexes. The best 4 complexes and a substrate peptide were selected for 200 ns MD simulation based on the data analysis. The simulation was run using the YASARA^49^ Dynamics software at a temperature of 298 K with a Berendsen thermostat controlled, and the simulation speed was 1–1.25 fs. The AMBER 14 force field was used for the calculation, and NaCl salt (0.9%) and water molecules (0.998 g/cm^3^ density) were introduced to the system for neutralization. The Particle-mesh Ewald approach was used to calculate long-range electrostatic interactions. A cuboid simulation cell with dimensions 20 Å greater than the protein–peptide complex was constructed in accordance with periodic boundary requirements. Using a simulated annealing strategy, the system’s conformational stress was decreased using the steepest gradient method (5000 cycles). The MD trajectories were finally saved at 100 ps intervals for later examination.

### Binding Free Energy Calculation

Binding free energy was calculated by the PRODIGY server considering 101 snapshots of the last 100 ns simulation.^50^ The binding free energy between a ligand (peptide) and a receptor (protein) to form a complex RL is predicted as
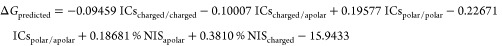
1

ICs_xxx/yyy_ means; Interfacial Contacts found at the interface between Interactor1 and Interactor2 classified according to the polar/apolar/charged nature of the interacting residues like ICs_charged/apolar_ is the number of ICs between charged and apolar residues. Two residues are said to be in contact if any of their heavy atoms are within 5.5 Å of one another. Based on the predicted binding affinity (Δ*G*) according to eq 1, the dissociation constant (*K*_d_) is calculated via the following eq 2:
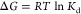
2where *R* denotes the ideal gas constant (in kcal K^–1^mol^–1^), *T* is the temperature (in K), and Δ*G* is the predicted free energy, and the temperature is set at 298.15 K (25.0 °C).

### Principal Component Analysis (PCA)

Analyzing by principal components, the hidden structural and energy profile within different groupings can be revealed via PCA analysis,^51,52^ here, considering the structural and energy information, such as the bonding distances, bond angles, dihedral angles, planarity, van der Waals energies, and electrostatic energies. PCA analysis used the final 50 ns of MD trajectory data for both peptide–protein complexes and NS2B/NS3–Apo complex.

The multivariate factors were placed in the *X* matrix and reduced into an outcome of two new matrices by using the eq 3 given below;
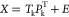
3Here, data matrix, *X* is the outcome of two new matrices i.e., *T*_k_ and *P*_k_^T^, *T*_k_ is the matrix of scores that shows how the sample relates to each other, *P*_k_ is the matrix of loadings that shows how the variables are related to each other, k is the total number of factors in the model, and *E* is the unmodeled variance.

### Peptide Structure–Activity Relationship Analysis

Peptide structure–activity relationship (SAR) analysis is a widely used method for investigating the correlation between the structure of a peptide and its biological activity. In this study, the top 30 peptides were selected for SAR analysis to investigate the binding affinity of these peptides to the Zika virus NS2B/NS3 protease. To determine the properties of the selected peptides, various computational tools were utilized, including ProtParam, Peptide Property Calculator, gravy calculator, and PepCalc.^53−55^ These tools were used to calculate various peptide characteristics, such as amino acid composition, molecular weight, approximate volume, gravity, and net charge at pH 7, and some other general properties are also used in SAR calculation like acidic (A), basic (B), aromatic (AR), non-polar (NP), polar (P) amino acids. Initially, sequential multiple linear regression analysis was then performed using these peptide characteristics to estimate the binding affinities of the test peptides to the Zika virus NS2B/NS3 protease. The four most important peptide characteristics were further analyzed using principal component analysis (PCA) to group the peptides in a biplot and investigate the structural variance.

## Supporting Information Available

Sequence, length, inhibition efficiency, binding affinity, average of Vdw, angle, bond, dihedral, coulomb parameters (PDF)
