# Supplementary material for: Therapeutic Potential of Antiviral Peptides against the NS2B/NS3 Protease of Zika Virus
Source: ACS Omega. 2023 Sep 13;8(38):35207–18. doi: 10.1021/acsomega.3c04903 (PMC10536883; doi:10.1021/acsomega.3c04903)
Supplement: Supplementary file 1 — ao3c04903_si_001.pdf [file ao3c04903_si_001.pdf]

Therapeutic Potential of Antiviral Peptides against the NS2B/NS3 Protease of Zika virus

Md. Shahadat Hossain[1$], Md. Tanjil Islam Shovon[1$], Md. Rafid Hasan[1$], Fuad Taufiqul Hakim[1$], Mohammad Mehedi Hasan[1], Sadia Afrose Esha[1], Sabiha Tasnim[1], Md. Shahoriar Nazir[1], Fahmida Akhter[1], Md Ackas Ali[2], Mohammad A. Halim[2*]

*Correspondence to: Mohammad A. Halim[2] (E-mail: mhalim1@kennesaw.edu)

[1] Division of Infectious Diseases and Division of Computer-Aided Drug Design, The Red-Green Research Center, BICCB, Tejgaon-1215, Dhaka, Bangladesh

[2] Department of Chemistry and Biochemistry, Kennesaw State University, Kennesaw, GA 30144, USA

[$]These authors contributed equally.

Table S1. Sequence, length, potency of the 429 antiviral peptides against various cell lines infected with Zika Virus (retrieved from AVPdb).

Peptide ID	Sequence	Length	Cell Line	Inhibition/ IC50	Unit
**Replication**					
AVP0003	ECRSTSYAGAVVNDL	15	BHK-21/C	42	µM
AVP0004	STSYAGAVVNDL	12	BHK-21/C	29	µM
AVP0006	AGAVVNDL	8	BHK-21/C	283	µM
AVP0007	GAVVNDL	7	BHK-21/C	225	µM
AVP0008	AVVNDL	6	BHK-21/C	190	µM
AVP0009	VVNDL	5	BHK-21/C	760	µM
AVP0010	YAVVNDL	7	BHK-21/C	340	µM
AVP0012	AAGAVVNDL	9	BHK-21/C	280	µM
AVP0013	YAGAVANDL	9	BHK-21/C	760	µM
AVP0016	GQPEEGAPCQVVLQGA	16	NA	3.5	µM
AVP0017	RGILIHNTIFGEQVF	15	NA	7.5	µM
AVP0018	YRWRGPTAAFLSLV	14	NA	9	µM
AVP0019	SSSTSTQVQILSNAL	15	NA	0.8	µM
AVP0160	VETTVTTAQETKRGRIQTK K	20	A9	High	NA
AVP0161	RPETVETTVTTAQETKRGRI	20	A9	NA	NA
AVP0162	VTTAQETKRGRIQTKKEVSI	20	A9	NA	NA
AVP0176	LWVGGGRNA	9	Grass carp kidney cells	10^-6.0	TCI D50
AVP0460	YDHIQDHVNTMFSRLATSW CLLQNKERALWAEAA	34	MDBK	5	µM
AVP0623	RTQRRGRTGRGKPGIYR	17	Vero	27.1±2.4	µM
AVP0628	RGIYR	5	Vero	>500	µM
AVP0629	SQVGD	5	Vero	>500	µM
AVP0635	RNPNQVGD	8	Vero	>500	µM
AVP0641	RRGRTGRGRRGIYR	14	Vero	2.7±0.3	µM
AVP0642	RTGRGRRGIYR	11	Vero	106±6.1	µM
AVP0643	RGRRGIYR	8	Vero	397±10.6	µM
AVP0646	STQRRGRTGRGRRGIYR	17	Vero	47.2±4.7	µM
AVP0653	RIGRNPSQVGD	11	Vero	285±8.1	µM
AVP0657	RNPSQVGD	8	Vero	437±13.4	µM
AVP0659	RRGRIGRNPSQVGD	14	Vero	191±7.3	µM
AVP0660	AAQRRGRIGRNPSQVGD	17	Vero	>500	µM
AVP0663	RVGRNPNQVGD	11	Vero	448±14.3	µM
AVP0666	NQVGD	5	Vero	>500	µM

AVP0669	RRGRVGRNPNQVGD	14	Vero	253±6.5	µM
AVP0670	AAQRRGRVGRNPNQVGD	17	Vero	>500	µM
AVP0874	RQLVLRTR	8	Flp-In-293	75	%
AVP0875	EALMWEGF	8	Flp-In-293	40	%
AVP0970	SFYSVLFLWGTCGGFSHSW Y	20	HepG2	58.1	%
AVP0971	CNDFRSKTC	9	MDCK	53	%
AVP0973	SSCNMGWDTPACCVWFPY WV	20	HeLa	70	%
AVP0974	MAVGLVLCDWWLGEYLLE A	19	HeLa	70	%
AVP0975	PVLQPALSLSCGPEPLLLSC	20	HeLa	40	%
AVP0976	IEVTFVNRRGDGAELWYLS A	20	HeLa	35	%
AVP0977	MDVNPTLLFLKVPAQNAIS TTFPYT	25	MDCK	1.8	nM
AVP0978	VNPTLLFLKVPAQNAISTTF PYT	23	MDCK	661.77	nM
AVP0979	PTLLFLKVPAQNAISTTFPY T	21	MDCK	483	nM
AVP0980	LLFLKVPAQNAISTTFPYT	19	MDCK	>3000	nM
AVP0981	FLKVPAQNAISTTFPYT	17	MDCK	>3000	nM
AVP0982	KVPAQNAISTTFPYT	15	MDCK	>3000	nM
AVP0983	MDVNPTLLFLKVPAQNAIS T	20	MDCK	33.8	nM
AVP0984	MDVNPTLLFLKVPAQNAI	18	MDCK	29.45	nM
AVP0985	MDVNPTLLFLKVPAQN	16	MDCK	45.86	nM
AVP0987	MDVNPTLLFLKVPA	14	MDCK	34.53	nM
AVP0988	MDVNPTLLFLKVP	13	MDCK	138.17	nM
AVP0989	MDVNPTLLFLKV	12	MDCK	644	nM
AVP0990	MDVNPTLLFLK	11	MDCK	899	nM
AVP0991	MDVNPTLLFL	10	MDCK	>3000	nM
AVP0992	MDVNPTLLF	9	MDCK	>3000	nM
AVP0993	MDVNPTLL	8	MDCK	>3000	nM
AVP0994	MDVNPTL	7	MDCK	>3000	nM
AVP0995	MDVNPT	6	MDCK	>3000	nM
AVP0996	MNINPYPLFIDVPIQ	15	MDCK	>3000	nM
AVP0997	MNINPTLLFLKVPIQ	15	MDCK	6.69	nM
AVP0998	MDVNPTLLFIDVPAQ	15	MDCK	>3000	nM
AVP0999	MNINPTLLFLKVPAQ	15	MDCK	12.96	nM
AVP1002	MDVNPYLLFLKVPAQ	15	MDCK	21.64	nM
AVP1003	MDVNPFLLFLKVPAQ	15	MDCK	2.84	nM

AVP1004	MDVNPWLLFLKVPAQ	15	MDCK	3.4	nM
AVP1007	MDVNPTLLFLKVPAQ	15	MDCK	>3000	nM
AVP1012	MDVNPYFLFLKVPAQ	15	MDCK	345	nM
AVP1013	MDVNPTFLFLKVPAQ	15	MDCK	>3000	nM
AVP1017	MDVNPHLLFLKVPAQ	15	MDCK	>3000	nM
AVP1018	MDVNPCLLFLKVPAQ	15	MDCK	>3000	nM
AVP1060	MSTNPKPQRKTKRNTNRRPQDVKFPGGGQIVGGVYL	36	Huh7	High	NA
AVP1061	MSTNPKPQRKTKRNTNRRPQDVKFPGGGQIVGGVY	35	Huh7	NA	NA
AVP1062	MSTNPKPQRKTKRNTNRRPQDVKFPGGGQIVGGV	34	Huh7	NA	NA
AVP1063	TKRNTNRRPQDVKFPGGGQIVGGVYL	26	Huh7	High	NA
AVP1064	NRRPQDVKFPGGGQIVGGVYL	21	Huh7	High	NA
AVP1065	DVKFPGGGQIVGGVYL	16	Huh7	NA	NA
AVP1066	NRRPQDVKFPGGGQIVGGVY	20	Huh7	NA	NA
AVP1067	NRRPQDVKFPGGGQIVGGV	19	Huh7	NA	NA
AVP1118	ASLRVRIKKQ	10	Vero	0.21±0.04	EC50 (µM)
AVP1119	ASLRVRIKK	9	Vero	0.25±0.01	EC50 (µM)
AVP0020	RRLQVGGGTLKFFLT	15	NA	12	µM
AVP1199	CGYKLC	6	NA	16.7	µM
AVP1200	CGYGLC	6	NA	15.1	Ki (µM)
AVP1202	CGYKGC	6	NA	12	Ki (µM)
AVP1203	CGYKRC	6	NA	5	Ki (µM)
AVP1204	CGKRRC	6	NA	3.5	Ki (µM)
AVP1205	CGKRKLC	7	NA	6.7	µM
AVP1206	CGKRKSC	7	NA	3.3	µM
AVP1207	AGKRKSG	7	NA	16.7	µM
AVP1208	CAGKRKSG	8	NA	6.7	µM
AVP1211	GELGRLVYLLDGPGYDPIHCSLAYGDASTLVVF	33	E. coli	20.7±3.6	nM
AVP1212	GELGRLVYLLDGPGYDPI	18	E. coli	125.4	nM

AVP1213	HCSLAYGDASTLVVF	15	E. coli	>>1	nM
AVP1214	GELGRPVYVLGDPGYYATH CIYATTNDALIFSV	33	E. coli	25.6±3.5	nM
AVP1215	GELGRPVYVLGDPGYYAT	18	E. coli	227.6	nM
AVP1216	HCIYATTNDALIFSV	15	E. coli	>>1	nM
AVP1217	GELGRIPSDTYDLAVGALH CPFYLVSGLVYLDG	33	E. coli	>>1	nM
AVP1218	GELGRLVYLLDGPGYDPIH CDVVTRGGSHLFNF	33	E. coli	11.3±1.6	nM
AVP1219	GELDELVYLLDGPGYDPIH CDVVTRGGSRLFNF	33	E. coli	1.14±0.053	nM
AVP1220	GELGRLVYLLDGPGYDPIH CD	21	E. coli	124	nM
AVP1221	GELDELVYLLDGPGYDPIHS	20	E. coli	22.8	nM
AVP1222	RQIKINFQNRRMKNKKGEL DELVYLLDGPGYDPIHS	36	E. coli	23.6	nM
AVP1236	ESGRIKKEEFAEIMKICSTIE ELRRQK	27	HEK293T	High	NA
AVP1251	YPYYPGEARGAP	12	CV1	1000	fold
AVP1703	AKVTMTCSAS	10	LLC-MK2	2.686x10^-6	mol/ liter
AVP1715	EVVLFLLNVF	10	MRC-5	5.9±0.2	log10 TCID50/ ml
AVP1722	FGGASCCLYCRCHIDHPNP KGFCDLKGKY	29	E. coli	160	µM
AVP1723	GGASCCLYCRCH	12	E. coli	160	µM
AVP1724	DLKGKYVQIP	10	E.coli	Low	µM
AVP1725	NCVKMLCTHTGTGQAITVT P	20	E.coli	Low	µM
AVP1726	PTTCANDP	8	E.coli	Low	µM
AVP1727	HRILMRIRAMMT	12	MARC-145	34.13563	µM
AVP1728	HRALMRIRQMMT	12	MARC-145	34.18095	µM
AVP1729	HRILMRIR	8	MARC-145	43.50202	µM
AVP1730	HRIAMRIRQMMT	12	MARC-145	54.5872	µM
AVP1731	HRILMRIRQMMT	12	MARC-145	56	µM
AVP1732	ARILMRIRQMMT	12	MARC-145	56.12263	µM
AVP1733	HRILMRIRQAMT	12	MARC-145	57.98146	µM
AVP1734	HRILMRIAQMMT	12	MARC-145	60.60846	µM
AVP1737	HRILMRIRQMMA	12	MARC-145	89.41235	µM
AVP1738	HRILMRIRQMAT	12	MARC-145	120.5239	µM

AVP1739	HRILMRARQMMT	12	MARC-145	150.1405	μM
AVP1740	LMRIRQMMT	9	MARC-145	263.8101	μM
AVP1741	HRILARIRQMMT	12	MARC-145	435.4996	μM
AVP1742	LMRIR	5	MARC-145	716.4193	μM
AVP1743	HAILMRIRQMMT	12	MARC-145	NA	μM
AVP1744	HRILMAIRQMMT	12	MARC-145	NA	μM
AVP1748	FFLLFLQGAAGNSVLCRIRG GRCHVGSCHFPERHIGRCS GFQACCIRTWG	50	Duck embryo	High	NA
AVP1749	MQILPLLFAVLLLMLQAEP GLSLARGLPQDCERRGGFC SHKSCPPGIGRIGLCSKEDF CCRSRWYS	66	Duck embryo	High	NA
AVP1750	MRILYLLLSVLFVVLQGVA GQPYFSSPIHACRYQRGVCI PGPCRWPYYRVGSCGSGLK SCCVRNRWA	67	Duck embryo	High	NA
AVP1751	MRIVYLLFPFILLLVQGAAG SSLAPRNKEKCHREKGFCG FLKCFPFIISGKCSRFFFCCK KIFG	64	Duck embryo	High	NA
AVP1752	TATQCRIRGGFCRVGSCRFP HIAIGKCATFISCCGRAYEV DALNSVRTSPWLLAPGNNP H	60	Duck embryo	High	NA
AVP1811	GICRCICGRGICRCICGRIGG RVPGVGVPGVGHHHHHH	38	Vero	21.4±1.6	μM
AVP1821	RGGRLCYCRRRFCVCVGR	18	MK2	11.7	μM
AVP1852	GRTTLSTRGPPRGGPG	16	HeLa	High	NA
AVP1922	LLGDFFRKSKEKIGKEFKRI VQRIKDFLRNLVPRTES	37	BSC-1	High	NA
AVP1924	TDVILMCFSIDSPDSLENI	19	HEp-2	7.6	μM
AVP1925	CSIELSDIPLSVDFNTMID	19	HEp-3	>50	μM
AVP1926	ADVILMCFSIDSPDSLENI	19	HEp-4	2.56	μM
AVP1927	TAVILMCFSIDSPDSLENI	19	HEp-5	1.37	μM
AVP1928	TDAILMCFSIDSPDSLENI	19	HEp-6	6.6	μM
AVP1929	TDVALMCFSIDSPDSLENI	19	HEp-7	11.6	μM
AVP1930	TDVIAMCFSIDSPDSLENI	19	HEp-8	5.42	μM
AVP1931	TDVILACFSIDSPDSLENI	19	HEp-9	1.43	μM
AVP1932	TDVILMAFSIDSPDSLENI	19	HEp-10	>50	μM
AVP1933	TDVILMCASIDSPDSLENI	19	HEp-11	6.29	μM
AVP1934	TDVILMCFAIDSPDSLENI	19	HEp-12	6.82	μM
AVP-1935	TDVILMCFSADSPDSLENI	19	HEp-2	3.52	μM

AVP1936	TDVILMCFSIASPDSLENI	19	HEp-2	4.36	µM
AVP1937	TDVILMCFSIDAPDSLENI	19	HEp-2	2.26	µM
AVP1938	TDVILMCFSIDSADSLENI	19	HEp-2	15.32	µM
AVP1939	TDVILMCFSIDSPASLENI	19	HEp-2	2.61	µM
AVP1940	TDVILMCFSIDSPDALENI	19	HEp-2	1.19	µM
AVP1941	TDVILMCFSIDSPDSAENI	19	HEp-2	2.27	µM
AVP1942	TDVILMCFSIDSPDSLANI	19	HEp-2	9.83	µM
AVP1943	TDVILMCFSIDSPDSLEAI	19	HEp-2	18.47	µM
AVP1944	TDVILMCFSIDSPDSLENA	19	HEp-2	4.89	µM
AVP1945	TDVILMCFSI	10	HEp-2	>50	µM
AVP1946	TDVILMCFSIDSP	13	HEp-2	>50	µM
AVP1947	TDVILMCFSIDSPDSL	16	HEp-2	10.86	µM
AVP1948	DVILMCFSIDSPDSLENI	18	HEp-2	1.23	µM
AVP1949	VILMCFSIDSPDSLENI	17	HEp-2	16.95	µM
AVP1950	ILMCFSIDSPDSLENI	16	HEp-2	7.17	µM
AVP1951	CFSIDSPDSLENI	13	HEp-2	>50	µM
AVP1952	ILMCFSIDSPDSLEN	15	HEp-2	1.75	µM
AVP1953	ILMCFSIDSPDSLE	14	HEp-2	3.5	µM
AVP1954	ILMCFSIDSPDSL	13	HEp-2	12.4	µM
AVP1955	ILMCFSIDSPDS	12	HEp-2	6.36	µM
AVP1956	ILMCFSIDSPD	11	HEp-2	4.61	µM
AVP1957	ILMCFSIDSP	10	HEp-2	35.77	µM
AVP1958	ILMCFSIDS	9	HEp-2	>50	µM
AVP1959	ILMCFSID	8	HEp-2	>50	µM
AVP1960	ILMCFSI	7	HEp-2	>50	µM
AVP1961	ILMCFS	6	HEp-2	>50	µM
AVP1967	NKSLLTEVETPIRNEWGCR CNDSSD	25	MDCK	High	NA
AVP1968	KSLLTEVETPIRGSLLTEVET PIR	24	MDCK	High	NA
AVP1969	KETPIRNEWGCRGETPIRNE WGCR	24	MDCK	Low	NA
AVP1970	KNEWGCRCNDSSDGNEWG CRCNDSSD	26	MDCK	Low	NA
AVP1973	QLQKWEDWVRWIGNIPQY LKG	21	MBM	0.03	µg/m l
AVP1974	QKWEDWVRWIGN	12	MBM	0.25	µg/m l
AVP1975	WEDWVRWIGNIP	12	MBM	0.04	µg/m l
AVP1976	QLQKWEDWVRWI	12	MBM	0.03	µg/m l

AVP1977	WEDWVRWI	8	MBM	0.06	µg/ml
AVP1978	DWVRWI	6	MBM	0.15	µg/ml
AVP1979	WEDWVR	6	MBM	>50	µg/ml
AVP1980	WVRWI	5	MBM	0.2	µg/ml
AVP1983	AEDWVRWI	8	MBM	4.30±0.70	µg/ml
AVP1984	WADWVRWI	8	MBM	0.16±0.03	µg/ml
AVP1985	WEAWVRWI	8	MBM	0.10±0.02	µg/ml
AVP1986	WEDAVRWI	8	MBM	>50	µg/ml
AVP1987	WEDWARWI	8	MBM	0.07±0.03	µg/ml
AVP1988	WEDWVAWI	8	MBM	0.09±0.03	µg/ml
AVP1989	WEDWVRAI	8	MBM	>50	µg/ml
AVP1990	WEDWVRWA	8	MBM	0.15±0.01	µg/ml
AVP2021	SMEKLAGFGAVGAGATAEETRRMLHRAFDTLA	32	Vero	15	µM
AVP2028	CATCEQIADSQHRSHRQMV	19	MDCK	0.7	nM
AVP2030	YAGAVVNDL	9	BHK-21/C13	52	µM
AVP2031	YAGAVVNDLL	10	BHK-21/C13	28	µM
AVP2032	YGAVVNDL	8	BHK	21	µg
AVP2035	LGNWAREIWATL	12	CrFK	High	NA
AVP2037	ANVVATYPAHS	11	Buffalo Green	5	µM
AVP2038	YLTTLK	6	HT-29	1000±25	µg/ml (EC50)
AVP2040	KAAKKAAKAAKKAAKWAKKAA	21	MRC-5	117	µM
AVP2041	AKKAAKKAKKAAKKAKKAAKK	21	MRC-5	41	µM
AVP2042	AKKAAKKAKKAAKKAKKWAKK	21	MRC-5	47.3	µM
AVP2043	AKKAWKKAKKAAKKAKKWAKK	21	MRC-5	40.8	µM

AVP2044	ARRAWRRARRAARRARRA ARR	21	MRC-5	18.2	µM
AVP2045	ARRAKRRARRAARRARRK ARR	21	MRC-5	13	µM
AVP2046	ARRAKRRARRAKRRARRK KRR	21	MRC-5	24.6	µM
AVP2050	LFRLIKSLIKRLVSAFK	17	Vero	No	NA
AVP2053	LFGLIPSLIGGLVSAFK	17	MDCK	7.12	µM(EC50)
AVP2055	SLIGGLVSAFK	11	MDCK	No	NA
AVP2060	VSAFK	5	MDCK	No	NA
**NS3 Protease**					
AVP0222	DDSVVCAAMSYSYA	14	BL21(DE3)	22	%
AVP0223	ADLEVVAATYVLVA	14	BL21(DE3)	33	%
AVP0224	KKKKVVAGTYVLV	13	BL21(DE3)	30	%
AVP0225	KKKKVVAAGYVLV	13	BL21(DE3)	2	%
AVP0226	KKKKVVAATGVLV	13	BL21(DE3)	6	%
AVP0227	KKKKVVAATYGLV	13	BL21(DE3)	0	%
AVP0228	KKKKVVAATYVGV	13	BL21(DE3)	14	%
AVP0229	KKKKVVAATYVLG	13	BL21(DE3)	13	%
AVP0230	KKKKVVAATYVLV	13	BL21(DE3)	17	%
AVP0231	KKKKPVAATYVLV	13	BL21(DE3)	24	%
AVP0232	KKKKVPAATYVLV	13	BL21(DE3)	3	%
AVP0233	KKKKVVAAPYVLV	13	BL21(DE3)	0	%
AVP0234	ADLEVVAATFVLVA	14	BL21(DE3)	49	%
AVP0235	KKKKVVAPTYVLV	13	BL21(DE3)	0	%
AVP0236	KKKKVVAATPVLV	13	BL21(DE3)	0	%
AVP0237	KKKKVVAATYPLV	13	BL21(DE3)	0	%
AVP0238	KKKKVVAATYVPV	13	BL21(DE3)	10	%
AVP0239	KKKKVVAATYVLP	13	BL21(DE3)	0	%
AVP0240	KKKKLLLPFLFFV	13	BL21(DE3)	0	%
AVP0241	KKKKVVLATLVLV	13	BL21(DE3)	5	%
AVP0242	KKKKLLLPFLFLV	13	BL21(DE3)	0	%
AVP0243	KKKKVLLPFLFFV	13	BL21(DE3)	0	%
AVP0244	KKKKLVLPFLFFV	13	BL21(DE3)	6	%
AVP0245	ADLEVVAATHVLVA	14	BL21(DE3)	29	%
AVP0246	KKKKLLAPFLFFV	13	BL21(DE3)	4	%
AVP0247	KKKKLLLAFLFFV	13	BL21(DE3)	4	%
AVP0248	KKKKLLLPTLFFV	13	BL21(DE3)	2	%

AVP0249	KKKKLLLPFYFFV	13	BL21(DE3)	0	%
AVP0250	KKKKLLLPFLVFV	13	BL21(DE3)	0	%
AVP0251	KKKKLLAATYVLV	13	BL21(DE3)	0	%
AVP0252	KKKKLVLATYVLV	13	BL21(DE3)	2	%
AVP0253	KKKKLVAPTYVLV	13	BL21(DE3)	0	%
AVP0254	KKKKLVAAFYVLV	13	BL21(DE3)	18	%
AVP0255	KKKKLVAATYVFV	13	BL21(DE3)	8	%
AVP0256	ADLEVVAATYV	11	BL21(DE3)	15	%
AVP0257	KKKKLVAATYVLF	13	BL21(DE3)	14	%
AVP0258	KKKKVLLATYVLV	13	BL21(DE3)	0	%
AVP0259	KKKKVLAPTYVLV	13	BL21(DE3)	1	%
AVP0260	KKKKVLAAFYVLV	13	BL21(DE3)	0	%
AVP0261	KKKKVLAATLVLV	13	BL21(DE3)	0	%
AVP0262	KKKKVLAATYFLV	13	BL21(DE3)	0	%
AVP0263	KKKKVLAATYVFV	13	BL21(DE3)	0	%
AVP0264	KKKKVVLPTYVLV	13	BL21(DE3)	0	%
AVP0265	KKKKVVLAFYVLV	13	BL21(DE3)	10	%
AVP0266	KKKKVVLATYVLF	13	BL21(DE3)	0	%
AVP0267	DLEVVAATYV	10	BL21(DE3)	0	%
AVP0268	KKKKVVAPFYVLV	13	BL21(DE3)	2	%
AVP0269	KKKKVVAPTLVLV	13	BL21(DE3)	3	%
AVP0270	KKKKVVAPTYFLV	13	BL21(DE3)	15	%
AVP0271	KKKKVVAPTYVFV	13	BL21(DE3)	0	%
AVP0272	KKKKVVAPTYVLF	13	BL21(DE3)	15	%
AVP0273	KKKKVVAAFLVLV	13	BL21(DE3)	20	%
AVP0274	KKKKVVAAFYFLV	13	BL21(DE3)	7	%
AVP0275	KKKKVVAAFYVFV	13	BL21(DE3)	0	%
AVP0276	KKKKVVAAFYVLF	13	BL21(DE3)	8	%
AVP0277	KKKKVVAATLFLV	13	BL21(DE3)	0	%
AVP0279	KKKKVVAATLVFV	13	BL21(DE3)	0	%
AVP0280	KKKKVVAATLVLF	13	BL21(DE3)	9	%
AVP0281	KKKKVVAATYFFV	13	BL21(DE3)	0	%
AVP0282	KKKKVVAATYVFF	13	BL21(DE3)	0	%
AVP0283	KKKKVLLPFLFFF	13	BL21(DE3)	2	%
AVP0284	KKKKLVLPFLFFF	13	BL21(DE3)	12	%
AVP0285	KKKKLLAPFLFFF	13	BL21(DE3)	14	%
AVP0286	KKKKLLLAFLFFF	13	BL21(DE3)	16	%
AVP0287	KKKKLLLPTLFFF	13	BL21(DE3)	0	%
AVP0288	KKKKLLLPFYFFF	13	BL21(DE3)	24	%

AVP0289	DDDEVVAATYVA	12	BL21(DE3)	0	%
AVP0290	KKKKLLLPFLVFF	13	BL21(DE3)	0	%
AVP0291	KKKKLLLPFLFLF	13	BL21(DE3)	4	%
AVP0292	KKKKLLLPFLFFF	13	BL21(DE3)	19	%
AVP0293	KKKKVVLPFLFFF	13	BL21(DE3)	29	%
AVP0294	KKKKLVAPFLFFF	13	BL21(DE3)	15	%
AVP0295	KKKKLLAAFLFFF	13	BL21(DE3)	30	%
AVP0296	KKKKLLLATLFFF	13	BL21(DE3)	16	%
AVP0297	KKKKLLLPTYFFF	13	BL21(DE3)	3	%
AVP0298	KKKKLLLPFYVFF	13	BL21(DE3)	0	%
AVP0299	KKKKLLLPFLVLF	13	BL21(DE3)	4	%
AVP0300	DDDEVVAATYVLVA	14	BL21(DE3)	1	%
AVP0301	KKKKVLAPFLFFF	13	BL21(DE3)	8	%
AVP0302	KKKKVLLAFLFFF	13	BL21(DE3)	0	%
AVP0303	KKKKVLLPFYFFF	13	BL21(DE3)	27	%
AVP0304	KKKKVLLPFLLFF	13	BL21(DE3)	0	%
AVP0305	KKKKVLLPFLFVF	13	BL21(DE3)	0	%
AVP0306	KKKKLVLAFLFFF	13	BL21(DE3)	18	%
AVP0307	KKKKLVLPTLFFF	13	BL21(DE3)	29	%
AVP0308	KKKKLVLPFYFFF	13	BL21(DE3)	14	%
AVP0309	KKKKLVLPFLLFF	13	BL21(DE3)	18	%
AVP0310	KKKKLVLPFLFVF	13	BL21(DE3)	40	%
AVP0311	ADLEVVAATYVDDD	14	BL21(DE3)	0	%
AVP0312	KKKKLLAPFLVFF	13	BL21(DE3)	9	%
AVP0313	KKKKLLLAFYFFF	13	BL21(DE3)	30	%
AVP0314	KKKKLLLAFLFLF	13	BL21(DE3)	8	%
AVP0315	KKKKLLLPTLVFF	13	BL21(DE3)	3	%
AVP0316	KKKKLLLPTLFLF	13	BL21(DE3)	0	%
AVP0317	KKKKLLLPFYFLF	13	BL21(DE3)	22	%
AVP0318	AESTTPCSGSYLKA	14	BL21(DE3)	0	%
AVP0319	EDCSTPSSGSFLDR	14	BL21(DE3)	0	%
AVP0320	KKKKVVTSTYVLVEA	15	BL21(DE3)	0	%
AVP0321	AKDLEVVTSTYVLVEA	16	BL21(DE3)	51	%
AVP0323	AKDLEVVCSTYVLVEA	16	BL21(DE3)	64	%
AVP0324	FDEMEESSSHLPYI	14	BL21(DE3)	0	%
AVP0325	AKDEMEECASHLPYEA	16	BL21(DE3)	0	%
AVP0326	DEMEEC	8	BL21(DE3)	0	%
AVP0327	DEMEECASHLPYK	15	BL21(DE3)	0	%
AVP0328	FDEMEECSQHLPYA	14	BL21(DE3)	0	%

AVP0329	FDEMEECASHLPYA	14	BL21(DE3)	0	%
AVP0330	AECVVSCSMSYTKA	14	BL21(DE3)	52	%
AVP0331	SEDVVSSSMSYTFT	14	BL21(DE3)	0	%
AVP0332	DEVVCC	8	BL21(DE3)	0	%
AVP0333	DDSVVCAAMSYSFA	14	BL21(DE3)	29	%
AVP0334	ADLEVVAATYVDVA	14	BL21(DE3)	54	%
AVP0335	ADLEVVAATYVLDA	14	BL21(DE3)	22	%
AVP0336	ADLEVVAATYVLVD	14	BL21(DE3)	38	%
AVP0337	ADLEVVAATYVDDA	14	BL21(DE3)	20	%
AVP0338	ADLEVVAATYVLDD	14	BL21(DE3)	44	%
AVP0339	ADLEVVAATYVDVD	14	BL21(DE3)	19	%
AVP0340	KKKKVVAATYVLVA	14	BL21(DE3)	0	%
AVP0341	KKKKVVAATYVKKA	14	BL21(DE3)	13	%
AVP0342	KKKKVVAATYVLKK	14	BL21(DE3)	0	%
AVP0343	KKKKVVAATYVLVK	14	BL21(DE3)	0	%
AVP0344	DDSVVCAAMSYSHA	14	BL21(DE3)	26	%
AVP0345	KKKKVVAATYFFFA	14	BL21(DE3)	5	%
AVP0346	KKKKVVAATYFLVA	14	BL21(DE3)	4	%
AVP0347	KKKKVVAATYVFVA	14	BL21(DE3)	0	%
AVP0348	KKKKVVAATYVLVF	14	BL21(DE3)	12	%
AVP0349	KKKKVVAATYVLFA	14	BL21(DE3)	12	%
AVP0350	KKKKVVAATYKKKA	14	BL21(DE3)	0	%
AVP0351	KKKKVVAATYKLKA	14	BL21(DE3)	0	%
AVP0352	KKKKVVAATYKKVA	14	BL21(DE3)	4	%
AVP0353	AKLKVVAATYVLKK	14	BL21(DE3)	5	%
AVP0354	AKLKVVAATYVKKK	14	BL21(DE3)	0	%
AVP0355	DDSVVSAAMSYSYA	14	BL21(DE3)	31	%
AVP0356	AKLEVVAATYVKKK	14	BL21(DE3)	0	%
AVP0357	AKLEVVAATYKKKK	14	BL21(DE3)	0	%
AVP0358	KKKKVVAATYKKKK	14	BL21(DE3)	8	%
AVP0359	ADKEVVAATYVKKK	14	BL21(DE3)	0	%
AVP0360	ADLEVVAATYVKKK	14	BL21(DE3)	2	%
AVP0361	ADLEVVAATYKKKK	14	BL21(DE3)	7	%
AVP0362	ADLKVVAATYVLVA	14	BL21(DE3)	0	%
AVP0363	ADLEVVAATYAAAA	14	BL21(DE3)	8	%
AVP0364	KDLKVVAATYVKKK	14	BL21(DE3)	21	%
AVP0365	KKKKAVAATYVLV	13	BL21(DE3)	59	%
AVP0366	DDSVVSAAMSYSFA	14	BL21(DE3)	14	%
AVP0367	KKKKVAAATYVLV	13	BL21(DE3)	32	%

AVP0368	KKKKVVAAAYVLV	13	BL21(DE3)	19	%
AVP0369	KKKKVVAATAVLV	13	BL21(DE3)	13	%
AVP0370	KKKKVVAATYALV	13	BL21(DE3)	0	%
AVP0371	KKKKVVAATYVAV	13	BL21(DE3)	21	%
AVP0372	KKKKVVAATYVLA	13	BL21(DE3)	19	%
AVP0373	KKKKLVAATYVLV	13	BL21(DE3)	0	%
AVP0374	KKKKVLAATYVLV	13	BL21(DE3)	12	%
AVP0375	KKKKVVLATYVLV	13	BL21(DE3)	32	%
AVP0376	KKKKVVALTYVLV	13	BL21(DE3)	0	%
AVP0377	DDSVVSAAMSYSHA	14	BL21(DE3)	0	%
AVP0378	KKKKVVAALYVLV	13	BL21(DE3)	7	%
AVP0379	KKKKVVAATYLLV	13	BL21(DE3)	26	%
AVP0380	KKKKVVAATYVLL	13	BL21(DE3)	25	%
AVP0381	KKKKFVAATYVLV	13	BL21(DE3)	30	%
AVP0382	KKKKVFAATYVLV	13	BL21(DE3)	26	%
AVP0383	KKKKVVFATYVLV	13	BL21(DE3)	34	%
AVP0384	KKKKVVAFTYVLV	13	BL21(DE3)	23	%
AVP0385	KKKKVVAAFYVLV	13	BL21(DE3)	3	%
AVP0386	KKKKVVAATFVLV	13	BL21(DE3)	28	%
AVP0387	KKKKVVAATYFLV	13	BL21(DE3)	14	%
AVP0388	DDSVVAAMSYSYA	13	BL21(DE3)	9	%
AVP0389	KKKKVVAATYVFV	13	BL21(DE3)	15	%
AVP0390	KKKKVVAATYVLF	13	BL21(DE3)	1	%
AVP0391	KKKKEVAATYVLV	13	BL21(DE3)	2	%
AVP0392	KKKKVEAATYVLV	13	BL21(DE3)	0	%
AVP0393	KKKKVVEATYVLV	13	BL21(DE3)	0	%
AVP0394	KKKKVVAETYVLV	13	BL21(DE3)	19	%
AVP0395	KKKKVVAAEYVLV	13	BL21(DE3)	5	%
AVP0396	KKKKVVAATEVLV	13	BL21(DE3)	4	%
AVP0397	KKKKVVAATYELV	13	BL21(DE3)	0	%
AVP0398	KKKKVVAATYVEV	13	BL21(DE3)	0	%
AVP0399	DDSVVAAMSYSFA	13	BL21(DE3)	2	%
AVP0400	KKKKVVAATYVLE	13	BL21(DE3)	0	%
AVP0401	KKKKKVAATYVLV	13	BL21(DE3)	0	%
AVP0402	KKKKVKAATYVLV	13	BL21(DE3)	25	%
AVP0403	KKKKVVKATYVLV	13	BL21(DE3)	40	%
AVP0404	KKKKVVAKTYVLV	13	BL21(DE3)	12	%
AVP0405	KKKKVVAAKYVLV	13	BL21(DE3)	0	%
AVP0406	KKKKVVAATKVLV	13	BL21(DE3)	0	%

AVP0407	KKKKVVAATYVKV	13	BL21(DE3)	0	%
AVP0408	KKKKVVAATYVLK	13	BL21(DE3)	0	%
AVP0409	KKKKTVAATYVLV	13	BL21(DE3)	27	%
AVP0410	DDSVVAAMSYSHA	13	BL21(DE3)	22	%
AVP0411	KKKKVTAATYVLV	13	BL21(DE3)	32	%
AVP0412	KKKKVVTATYVLV	13	BL21(DE3)	1	%
AVP0413	KKKKVVATTYVLV	13	BL21(DE3)	16	%
AVP0414	KKKKVVAATTVLV	13	BL21(DE3)	23	%
AVP0415	KKKKVVAATYTLV	13	BL21(DE3)	0	%
AVP0416	KKKKVVAATYVTV	13	BL21(DE3)	0	%
AVP0417	KKKKVVAATYVLT	13	BL21(DE3)	8	%
AVP0418	KKKKGVAATYVLV	13	BL21(DE3)	0	%
AVP0419	KKKKVGAATYVLV	13	BL21(DE3)	0	%
AVP0420	KKKKVVGATYVLV	13	BL21(DE3)	14	%
**Polymerase**					
AVP0149	CATCQIADSHRSHRQMV	17	MDCK	High	NA
AVP0150	CATCIADHRSHRQMV	15	MDCK	High	NA
AVP0151	CATCEQIADSQHRSHRQM	18	MDCK	Medium	NA
AVP0152	CATCEQIADSQHRSHRQ	17	MDCK	Medium	NA
AVP0153	CATCEQIADSQHRSHR	16	MDCK	Medium	NA
AVP0154	CATCEQIADSQHRSH	15	MDCK	Medium	NA
AVP0155	CATCQIADSHRSH	13	MDCK	Medium	NA
AVP0156	CATCIADHRSH	11	MDCK	Low	NA
AVP0157	CACIADHSH	9	MDCK	Low	NA
AVP0158	CTCIADHRH	9	MDCK	Low	NA
**RdRp**					
AVP0215	ACFPWGNTWCGGK	13	Huh7	150	µM
AVP0216	ACFPWGKEYCGGK	13	Huh7	22	µM
AVP0219	ACFPWGNQWCGGK	13	Huh7	37	µM
AVP0221	CFPWGC	6	Huh7	150	µM
**NS2/3**					
AVP0173	KGSVVIVGRIILSGRK	16	-	-	-
AVP0174	VRLGSISVIGIVRGKK	16	-	-	-
AVP0175	RGGSVVIVGRIILSGRK	17	-	-	-
**Substrate peptide**					
**Substrate peptide**	RKKR	4	-	-	-

Table S2. Binding scores of 429 peptides against the ZIKV NS2B/NS3 obtained from HawkDock and FireDock.

AVPid	HawkDock Score	Firedock Global Energy
**Replication**		
AVP0003	-34.59	-50.74
AVP0004	-28.15	-45.58
AVP0006	-19.51	-42.74
AVP0007	-29.3	-46.12
AVP0008	-12.1	-42.93
AVP0009	-10.5	-43.26
AVP0010	-41.45	-47.8
AVP0012	-18.72	-43.64
AVP0013	-35.94	-48.08
AVP0016	-21.94	-59.79
AVP0017	-27.62	-42.71
AVP0018	-69.08	-65.91
AVP0019	-51.84	-47.01
AVP0020	-42.92	-48.26
AVP0160	-38.58	-52.18
AVP0161	-30.29	-54.43
AVP0162	-44.3	-50.3
AVP0176	-31.19	-60.47
AVP0460	-49.18	-65.89
AVP0623	-68.53	-41.15
AVP0628	-38.31	-44.09
AVP0629	-20.12	-46.09
AVP0635	-27.12	-64.67
AVP0641	-64.78	-68.74
AVP0642	-57.22	-49.42
AVP0643	-51.91	-54.91
AVP0646	-42.55	-49.75
AVP0653	-33.31	-45.05
AVP0657	-28.13	-55.83
AVP0659	-38.23	-54.89
AVP0660	-60.08	-54.51
AVP0663	-42.19	-53.23
AVP0666	-18.96	-38.28
AVP0669	-52.71	-49.62

AVP0670	-41.61	-48.32
AVP0874	-43.15	-53.91
AVP0875	-29.15	-47.04
AVP0970	-41.69	-66.38
AVP0971	-28.66	-42.73
AVP0973	-31.54	**-72.81**
AVP0974	-34.3	-52.88
AVP0975	-38.33	-59.15
AVP0976	-33.65	-65.5
AVP0977	-42.43	-68.71
AVP0978	-38.02	-65.89
AVP0979	-22.62	-53.58
AVP0980	-35.83	-53.83
AVP0981	-31.82	-56.23
AVP0982	-34.83	-51.13
AVP0983	-34.67	-61.69
AVP0984	-31.55	-55.42
AVP0985	-44.91	-60.1
AVP0987	-47.08	-58.53
AVP0988	-22.19	-55.09
AVP0989	-16.06	-62.54
AVP0990	-27.73	-51.23
AVP0991	-31.83	-50.56
AVP0992	-24.01	-56.45
AVP0993	-23.98	-55.17
AVP0994	-25.23	-43.71
AVP0995	-11.3	-48.46
AVP0996	-22.86	-49.88
AVP0997	-32.19	-56.71
AVP0998	-33.81	-52.66
AVP0999	-28.91	-58.04
AVP1002	-47.99	-54.1
AVP1003	-40.84	-57.52
AVP1004	-31.41	-67.57
AVP1007	-25.18	-63.09
AVP1012	-29.99	-57.23
AVP1013	-36.89	-59.92
AVP1017	-25.12	-64.95
AVP1018	-31.32	-61.04
AVP1060	-55.79	-55.28

AVP1061	-56.41	-62.07
AVP1062	-34.82	-43.01
AVP1063	-41.95	-52.78
AVP1064	-35.29	-56.19
AVP1065	-33.66	-49.04
AVP1066	-32.08	-51.34
AVP1067	-37.87	-48.27
AVP1118	-46.65	-59.33
AVP1119	-52	-49.45
AVP1199	-31.08	-59.26
AVP1200	-8.28	-62.64
AVP1202	-27.68	-57.44
AVP1203	-34.78	-55.95
AVP1204	-41.1	-41.95
AVP1205	-41.48	-55.81
AVP1206	-44.97	-41.25
AVP1207	-47.25	-35.23
AVP1208	-46.29	-48.24
AVP1211	-25.02	-54.85
AVP1212	-43.8	-60.8
AVP1213	-43.6	-74.73
AVP1214	-34.24	-57.79
AVP1215	-14.33	-61.96
AVP1216	-37.29	-56.1
AVP1217	-51.41	-71.57
AVP1218	-25.71	-50.31
AVP1219	-13.55	-44.69
AVP1220	-47.3	-65.55
AVP1221	-15.74	-59.66
AVP1222	-25.48	-40.93
AVP1236	-46.49	-47.66
AVP1251	-31.26	-55.23
AVP1703	-28.05	-51.55
AVP1715	-18.3	-67.46
AVP1722	-24.48	-45.36
AVP1723	-39.15	-60.44
AVP1724	-27.02	-63.59
AVP1725	-35.61	-53.91
AVP1726	-4.53	-38.48
AVP1727	-38.83	-47.01

AVP1728	-47.2	-57.23
AVP1729	-55.35	-48
AVP1730	-46.22	-41.76
AVP1731	-26.58	-45.12
AVP1732	-37.05	-49.32
AVP1733	-43.71	-49.59
AVP1734	-36.79	-44.76
AVP1737	-28.29	-41.04
AVP1738	-38.99	-56.38
AVP1739	-40.49	-49.13
AVP1740	-38.11	-46.21
AVP1741	-49.77	-58.39
AVP1742	-37.81	-38.57
AVP1743	-34.14	-47.27
AVP1744	-32.92	-51.19
AVP1748	-56.56	-56.75
AVP1749	-24.22	-47.77
AVP1750	-37.01	-67.11
AVP1751	-38.37	-63.82
AVP1752	-61.98	-63.04
AVP1811	-51.88	-63.66
AVP1821	-58.53	-57.61
AVP1852	-35.12	-46
AVP1922	-38.56	-40.44
AVP1924	-28.93	-56.7
AVP1925	-27.43	-54.74
AVP1926	-12.75	-56.62
AVP1927	-13.09	-51.72
AVP1928	-25.48	-48.71
AVP1929	-28.78	-50.65
AVP1930	-33.1	-54.42
AVP1931	-32.13	-52.28
AVP1932	-27.18	-50.59
AVP1933	-26.94	-42.57
AVP1934	-8.04	-52.05
AVP1935	-15.41	-54.5
AVP1936	-21.64	-52.39
AVP1937	-24	-50.43
AVP1938	-10.06	-52.36
AVP1939	-28.51	-57.25

AVP1940	-9.67	-52.76
AVP1941	-35.64	-58.44
AVP1942	-2.17	-43.17
AVP1943	-40.22	-63.23
AVP1944	-23.42	-65.17
AVP1945	-26.59	-61.57
AVP1946	-17.6	-63.35
AVP1947	-30.7	-54.87
AVP1948	-20.39	-53.9
AVP1949	-24.86	-57.52
AVP1950	-5.17	-61.87
AVP1951	-26.44	-69
AVP1952	-26.78	-53.43
AVP1953	-37.11	-40.44
AVP1954	-32.26	-42.02
AVP1955	-20.55	-47.27
AVP1956	-24.08	-42.44
AVP1957	-30.87	-45.5
AVP1958	-28.51	-50.04
AVP1959	-24.96	-45.19
AVP1960	-20.57	-55.84
AVP1961	-29.54	-44.38
AVP1967	-26.49	-55.56
AVP1968	-24.11	-47.16
AVP1969	-42.59	-52.22
AVP1970	-42.05	-44.87
AVP1973	-29.53	-50.65
AVP1974	-29.85	-61.35
AVP1975	-41.77	-57.54
AVP1976	-27.19	-49.88
AVP1977	-32.31	-53.78
AVP1978	-28.97	-49.88
AVP1979	-20.37	-68.1
AVP1980	-30.41	-47.89
AVP1983	-30.91	-52.41
AVP1984	-18.02	-59.61
AVP1985	-40.8	-53.24
AVP1986	-21.39	-54.83
AVP1987	-38.36	-54.28
AVP1988	-21.52	-52.54

AVP1989	-37.52	-49.02
AVP1990	-29.85	-52.71
AVP2021	-24.43	-48.71
AVP2028	-39.99	-47.04
AVP2030	-37.16	-49.57
AVP2031	-34.11	-62.41
AVP2032	-25.81	-48.52
AVP2035	-38.85	-56.96
AVP2037	-25.58	-58.64
AVP2038	-52.6	-57.97
AVP2040	-29.87	-38.2
AVP2041	-30	-33.84
AVP2042	-37.91	-33.59
AVP2043	-34.73	-42.28
AVP2044	-85.17	-42.88
AVP2045	-67.1	-37.08
AVP2046	-77.2	-33.47
AVP2050	-34.86	-73.78
AVP2053	-29.92	-60.98
AVP2055	-32.67	-54.27
AVP2060	-24.59	-45.04
**NS3 Protease**		
AVP0222	-14.63	-57.9
AVP0223	-25.61	-63.24
AVP0224	-37.54	-46.33
AVP0225	-38.71	-60.49
AVP0226	-41.85	-61.5
AVP0227	-35.12	-52.91
AVP0228	-42.15	-59.83
AVP0229	-51.8	-61.51
AVP0230	-36.66	-58.62
AVP0231	-41.95	-52.33
AVP0232	-26.99	-45.3
AVP0233	-36.43	-59.85
AVP0234	-31.29	-52.86
AVP0235	-49.68	-61.44
AVP0236	-36.27	-58.11
AVP0237	-36.73	-63.07
AVP0238	-29.48	-48.41
AVP0239	-57.4	-62.62

AVP0240	-46.49	-65.94
AVP0241	-25.22	-57.5
AVP0242	-36.23	-53.52
AVP0243	-37.18	-55.62
AVP0244	-29.03	-66.15
AVP0245	-33.21	-54.42
AVP0246	-38.71	-66.15
AVP0247	-25.26	-64.87
AVP0248	-44.03	-50.3
AVP0249	-33.35	-60.05
AVP0250	-38.13	-52.92
AVP0251	-43.06	-49.76
AVP0252	-37.61	-58.87
AVP0253	-30.64	-45.43
AVP0254	-35.95	-60.22
AVP0255	-44.09	-61.89
AVP0256	-19.01	-52.28
AVP0257	-50.91	-56.6
AVP0258	-33.36	-59.25
AVP0259	-35.3	-49.05
AVP0260	-44.13	-67.39
AVP0261	-27.04	-48.92
AVP0262	-41.04	-65.09
AVP0263	-41.39	-58.25
AVP0264	-28.05	-60.6
AVP0265	-39.94	-54.88
AVP0266	-32.23	-47.07
AVP0267	-20.13	-52.07
AVP0268	-44.96	-59.23
AVP0269	-26.5	-66.49
AVP0270	-41.59	-52.42
AVP0271	-39.86	-57.95
AVP0272	-38.37	-46.96
AVP0273	-55.33	-73.37
AVP0274	-43.28	-52.37
AVP0275	-43.24	-69.47
AVP0276	-50.35	-68.97
AVP0277	-38.72	-62.89
AVP0279	-33.28	-52.75
AVP0280	-45.53	-47.09

AVP0281	-42.73	-51.98
AVP0282	-41.37	-63.89
AVP0283	-35.2	-63.18
AVP0284	-39.97	-51.67
AVP0285	-50.73	-54.53
AVP0286	-38.07	-52.15
AVP0287	-35.59	-56.11
AVP0288	-46.38	-51.17
AVP0289	-23.54	-61.13
AVP0290	-38.05	-68.79
AVP0291	-44.01	-62.27
AVP0292	-47.63	-68.21
AVP0293	-32.36	-63.46
AVP0294	-38.68	-57.64
AVP0295	-32.98	-59.33
AVP0296	-35.34	53.5
AVP0297	-31.35	-48.57
AVP0298	-40.53	-59.12
AVP0299	-43.22	-65.64
AVP0300	-15.42	-59.03
AVP0301	-42.01	-61.16
AVP0302	-42.79	-52.28
AVP0303	-36.07	-55.32
AVP0304	-42.56	-59.75
AVP0305	-43.89	-47.7
AVP0306	-40.3	-60.52
AVP0307	-39.49	-55.43
AVP0308	-29.51	-49.81
AVP0309	-43.65	-61.24
AVP0310	-43.11	-61.54
AVP0311	-27.23	-47.81
AVP0312	-42.41	-64.28
AVP0313	-50.33	-66.75
AVP0314	-37.45	-54.03
AVP0315	-36.66	-55.44
AVP0316	-49.32	-64.89
AVP0317	-40.98	-52.45
AVP0318	-39.08	-51.62
AVP0319	-26.95	-39.41
AVP0320	-25.32	-59.3

AVP0321	-49.83	-69.2
AVP0323	-40.77	-54.44
AVP0324	-27.33	-52.98
AVP0325	-12.74	-54.68
AVP0326	-27.85	-29.62
AVP0327	-29.97	-40.64
AVP0328	-28.73	-43.72
AVP0329	-13.41	-50.87
AVP0330	-23.1	-55.83
AVP0331	-36.85	-45.66
AVP0332	-24.22	-51.31
AVP0333	-26.63	-48.92
AVP0334	-20.82	-43.39
AVP0335	-20.01	-60.12
AVP0336	-22.52	-56.32
AVP0337	-24.66	-51.92
AVP0338	-15	-46.34
AVP0339	-28.43	-53.5
AVP0340	-46.86	-53.11
AVP0341	-26.5	-51.86
AVP0342	-42.13	-45.86
AVP0343	-44.61	-49.4
AVP0344	-35.88	-52.41
AVP0345	-40.99	-60.92
AVP0346	-43.69	-56.8
AVP0347	-46.26	-41.21
AVP0348	-47.27	-65.95
AVP0349	-43.8	-66.71
AVP0350	-32.71	-47.27
AVP0351	-36.59	-56.03
AVP0352	-38.11	-49.54
AVP0353	-29.77	-53.51
AVP0354	-36.5	-54.96
AVP0355	-34.84	-55.39
AVP0356	-20.08	-45.53
AVP0357	-40.95	-44.93
AVP0358	-39.08	-45.09
AVP0359	-19.7	-50.93
AVP0360	-24.63	-47.02
AVP0361	-27.67	-49.94

AVP0362	-13.51	-58.36
AVP0363	-35.52	-54.05
AVP0364	-38.18	-38.1
AVP0365	-52.6	-47.2
AVP0366	-10.28	-43.53
AVP0367	-40.98	-46.66
AVP0368	-49.84	-54.54
AVP0369	-36.51	-62.22
AVP0370	-37.29	-59.77
AVP0371	-36.27	-65.02
AVP0372	-52.09	-60.04
AVP0373	-52.5	-57.19
AVP0374	-34.13	-48.98
AVP0375	-41.23	-46.67
AVP0376	-49.11	-57.93
AVP0377	-28.57	-44.5
AVP0378	-52.44	-53.95
AVP0379	-34.91	-53.9
AVP0380	-51.37	-61.68
AVP0381	-34.58	-47.48
AVP0382	-51.08	-53.67
AVP0383	-35.32	-50.55
AVP0384	-37.01	-55.51
AVP0385	-52.2	-70.54
AVP0386	-38.33	-64.19
AVP0387	-42.56	-55.38
AVP0388	-34.04	-47.69
AVP0389	-37.99	-59.37
AVP0390	-55.05	-64.01
AVP0391	-33.03	-57.13
AVP0392	-43.81	-50.27
AVP0393	-32.66	-62.53
AVP0394	-37.07	-47.83
AVP0395	-50.32	-53.33
AVP0396	-35.84	-52.46
AVP0397	-39.48	-51.39
AVP0398	-31.08	-52.62
AVP0399	-36.34	-53.02
AVP0400	-47.75	-58.58
AVP0401	-38.19	-47.05

AVP0402	-40.07	-58.52
AVP0403	-42.53	-59.22
AVP0404	-48.69	-52.62
AVP0405	-52.49	-46.6
AVP0406	-50.8	-62.3
AVP0407	-39.53	-62.88
AVP0408	-51.92	-59.29
AVP0409	-37.43	-56.52
AVP0410	-11.99	-58.86
AVP0411	-22.84	-55.32
AVP0412	-38.25	-53.12
AVP0413	-49.72	-48.01
AVP0414	-37.36	-65.85
AVP0415	-40.83	-57.82
AVP0416	-43.11	-63.79
AVP0417	-49.76	-57.01
AVP0418	-31.51	-64.61
AVP0419	-42.29	-52
AVP0420	-18.64	-55.05
**Polymerase**		
AVP0149	-34.1	-48.26
AVP0150	-41.81	-42.43
AVP0151	-28.23	-38.71
AVP0152	-36.4	-41.37
AVP0153	-40.58	-32.87
AVP0154	-24.37	-34.99
AVP0155	-26.99	-42.04
AVP0156	-35.13	-50.17
AVP0157	-3.98	-52.41
AVP0158	-41.35	-49.54
**RdRp**		
AVP0215	-36.03	-56.67
AVP0216	-24.48	-52.25
AVP0219	-33.22	-47.77
AVP0221	-24.21	-53.42
**NS2/3**		
AVP0173	-42.26	-61.49
AVP0174	-43.43	-53.59
AVP0175	-38.81	-50.95
**Substrate peptide**		

**Substrate peptide**	**-39.68**	**Not available**

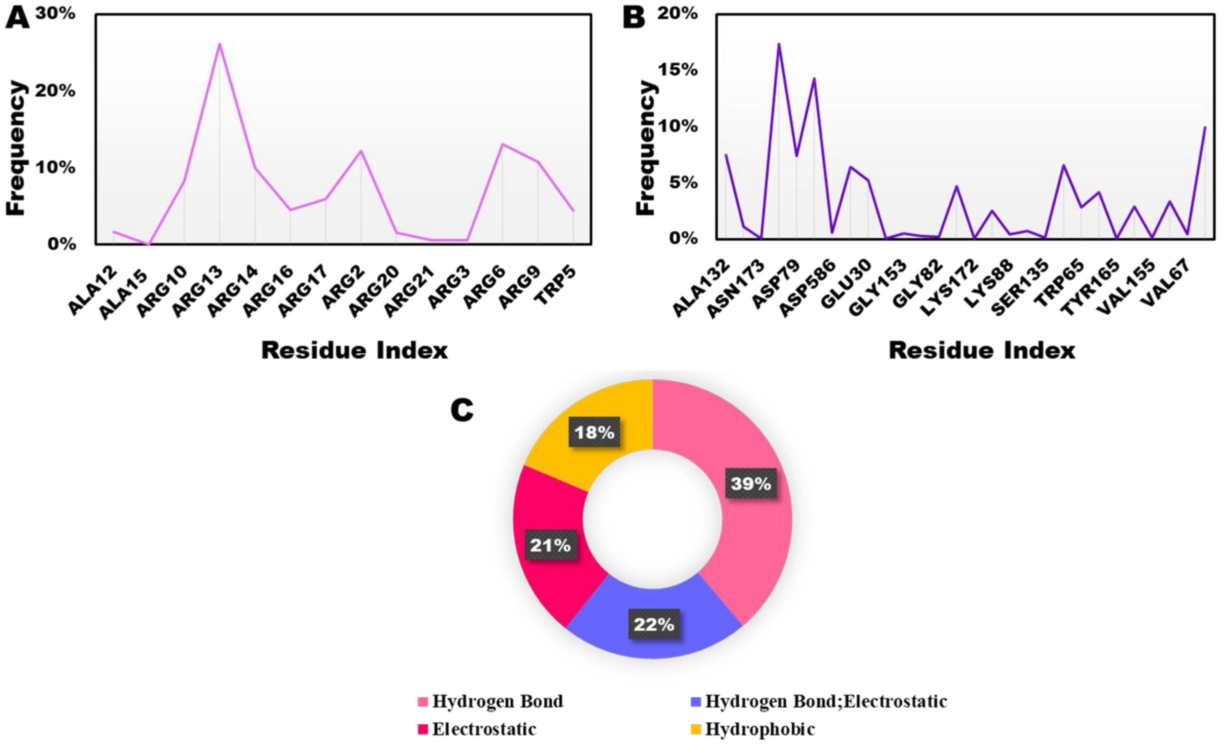

Figure S1. NS2B/NS3-AVP2044 complex. (A) Interacting AVP2044 residues; **(B)** Interacting NS2B/NS3 residues; **(C)** Distribution of non-covalent interactions; over 200 ns MD simulation.

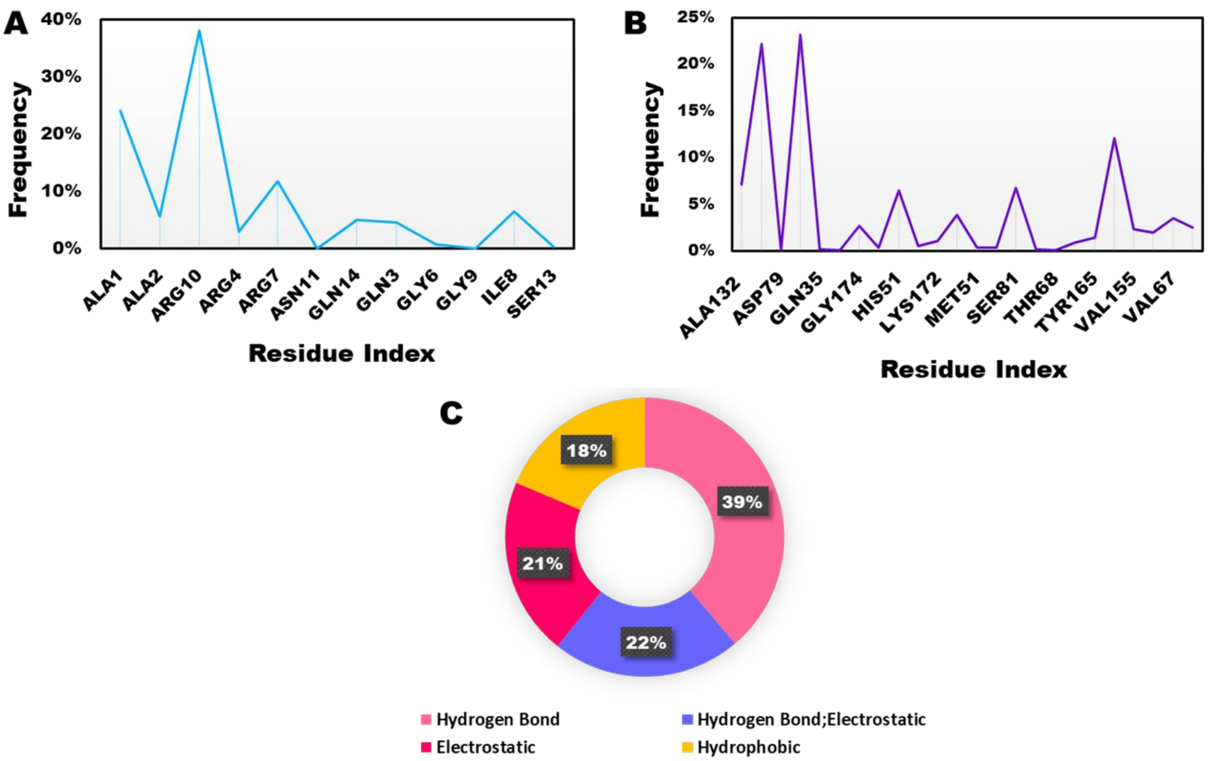

Figure S2. NS2B/NS3-AVP 0660 complex. (A) Interacting AYA9 residues; **(B)** Interacting NS2B/NS3 residues; **(C)** Distribution of non-covalent interactions; over 200 ns MD simulation.

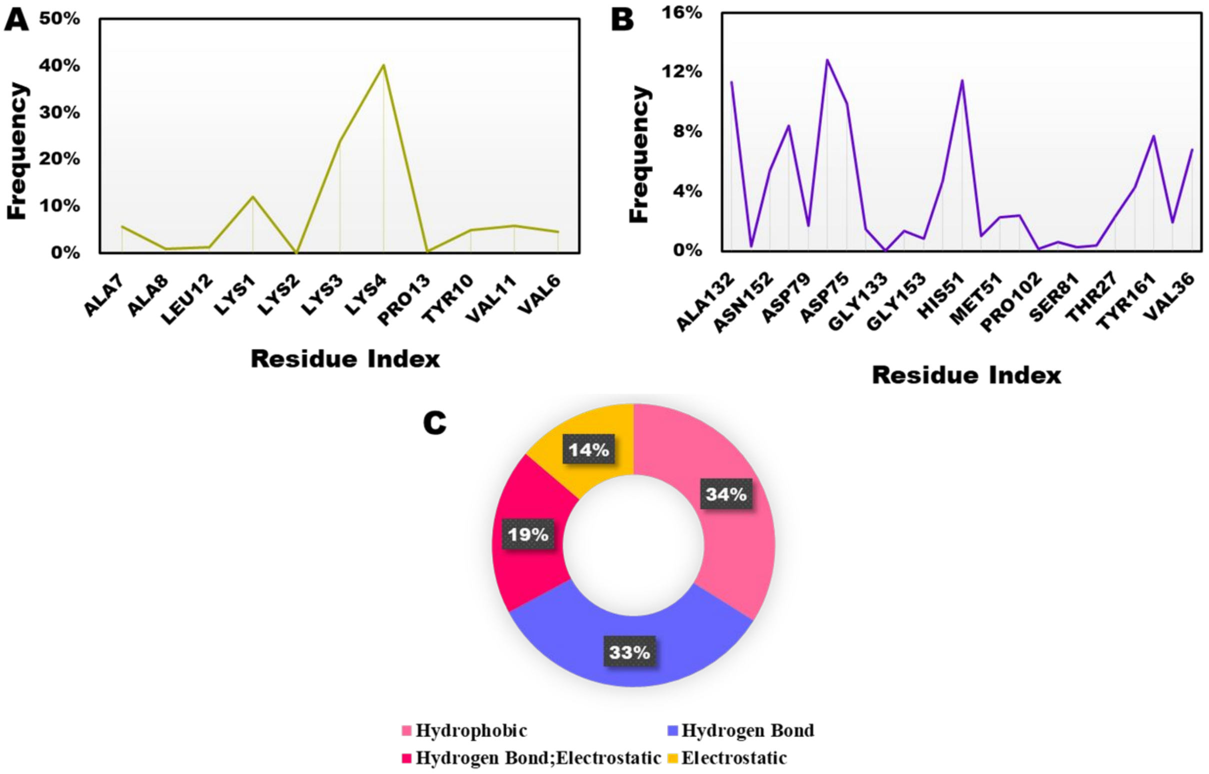

Figure S3. NS2B/NS3-AVP 0239 complex. (A) Interacting AYA9 residues; **(B)** Interacting NS2B/NS3 residues; **(C)** Distribution of non-covalent interactions; over 200 ns MD simulation.

Table S3: Stepwise MLR with most relevant peptide properties as predictors of HawkDock score of top 30 peptides.

**Predictors of HawkDock Score**	Step 1	Step 2	Step 3	Step 4	Step 5	Step 6
	(R^2 = 0.63)	(R^2 = 0.63)	(R^2 = 0.63)	(R^2 = 0.62)	(R^2 = 0.61)	(R^2 =0.6)
	**P Values**					
Approximate Volume	0.09990	0.07522	0.03362	0.00389	0.00181	1.82E-06
Non-Polar AA	0.27483	0.26065	0.04104	0.04470	0.02171	0.00014
Polar AA	0.29412	0.14435	0.10791	0.03989	0.00357	**0.00168**
GRAVY	0.33194	0.21248	0.25082	0.29911	**0.42825**	
No. of -Ve Charged AA	0.34668	0.28306	0.32784	**0.41369**		
No. of +Ve Charged AA	0.42438	0.38863	**0.56841**			
Net Charge at pH 7	0.52329	**0.51085**				
Theoretical pI	**0.94762**					

HawkDock Score = 2.226 Non-Polar AA + 1.45 Polar AA - 0.011 Approximate Volume - 51.25.

Table S4: Selected residues and their types in the contact Maps with NS2B/NS3

**Peptide ID**	**Sequence**
AVP0642<br>AA Type	R T G R G R R G I Y R<br>B P Np B Np B B Np Np Ar B
AVP2044<br>AA Type	A R R A W R R A R R A A R R A R R A A R R<br>Np B B Np Ar B B Np B B Np NP B B Np B B Np Np B B
AVP0239<br>AA Type	K K K K V V A A T Y V L P<br>B B B B Np Np Np Np P Ar Np Np Np
AVP0660<br>AA Type	A A Q R R G R I G R N P S Q V G D<br>Np Np P B B Np B Np Np B P Np P P Np Np A
